# Intensive Care Nurses' Self‐Reported Adherence to Evidence‐Informed Microaspiration‐Prevention Practices and Potential Implementation Determinants: A Multicenter Cross‐Sectional Study

**DOI:** 10.1002/nop2.70914

**Published:** 2026-10-21

**Authors:** Yongju Pei, Shuyu Fan, Xinping Shi

**Affiliations:** ^1^ Henan Provincial People's Hospital Zhengzhou City China; ^2^ Henan University of Chinese Medicine Zhengzhou City China

**Keywords:** adherence, implementation determinants, intensive care nurses, microaspiration, theoretical domains framework

## Abstract

**Aim:**

To assess intensive care nurses' self‐reported adherence to recommended microaspiration‐prevention practices and explore potential implementation determinants.

**Design:**

Multicentre, cross‐sectional.

**Methods:**

From December 2024 to February 2025, nurses in 31 intensive care units at 17 hospitals in Henan Province, China, completed a questionnaire on 22 preventive practices and 43 implementation‐related items informed by the Theoretical Domains Framework. Bivariate analyses, a fully adjusted three‐level mixed‐effects model accounting for unit and hospital clustering, and generalised estimating equation sensitivity analyses examined associations.

**Results:**

Of 510 respondents, 476 were analysed. Mean adherence was 77.17/110 (70.15% of maximum), with basic care scoring highest (73.6%) and risk management lowest (67.2%). After adjustment, adherence was lower in units with 10–14, 15–19 and ≥ 20 beds than in those with < 5 beds; the 5–9‐bed group did not differ. Both training exposures were associated with higher adherence in the primary analysis; only evidence‐based practice training remained significant in the 14‐item analysis. The lowest‐scoring items concerned near‐term implementation intention (3.10), competing priorities (3.21), and professional responsibility (3.29); the highest concerned time efficiency (4.19), workflow compatibility (4.15) and interest (3.94).

**Conclusion:**

Self‐reported adherence varied across practices and domains. Intensive care unit bed capacity and training exposure were associated with adherence, and implementation‐related findings identified potential areas for further investigation.

**Implications for Nursing Practice:**

Findings may guide intensive care unit nurses, nurse managers, and educators in strengthening targeted microaspiration‐prevention training, clarifying responsibilities, integrating preventive practices into routine workflows, and reinforcing bedside supervision, particularly in units with 10–14, 15–19, or ≥ 20 beds.

**Reporting Method:**

The Strengthening the Reporting of Observational Studies in Epidemiology guidelines were followed.

Patient or Public Contribution: None

## 1 Introduction

Microaspiration occurs when swallowing dysfunction caused by endotracheal intubation leads to the accumulation of oropharyngeal secretions above the tracheal cuff. These secretions gradually enter the lower respiratory tract because the cuff does not completely seal the trachea, making microaspiration a frequent complication in mechanically ventilated patients with a reported incidence of up to 88.9% (Liu et al. 2022). Microaspiration of oropharyngeal and subglottic secretions containing pathogenic microorganisms is considered a major pathogenic mechanism of ventilator‐associated pneumonia (VAP) (Blot et al. 2014; Bourgault et al. 2025; Millot et al. 2025). In Chinese intensive care units (ICUs), the incidence of VAP in mechanically ventilated patients is 30% (Li et al. 2024), with a mortality rate of 11.11% (Ding et al. 2022). Microaspiration and VAP can worsen the patient's condition, prolong mechanical ventilation, and increase ICU length of stay and medical costs.

Because microaspiration is an important upstream mechanism of VAP in ICU patients, its prevention may reflect the quality of nursing care to some extent. Although VAP prevention guidelines recommend core measures such as bedside elevation and cuff‐pressure monitoring (Chinese Medical Association‐Chinese Society of Critical Care Medicine 2013), VAP prevention has increasingly been addressed through multicomponent bundle initiatives and quality‐improvement programs. Evidence from a long‐term quality‐improvement study and a systematic review with meta‐analysis indicates that bundle‐based interventions can reduce VAP incidence and improve related clinical outcomes (Maertens et al. 2025; Martinez‐Reviejo et al. 2023).

However, these studies primarily demonstrate bundle‐level effects on VAP‐related outcomes (Maertens et al. 2025; Martinez‐Reviejo et al. 2023). Evidence remains limited regarding ICU nurses' adherence to microaspiration‐specific preventive practices targeting this upstream mechanism of VAP and the potential determinants of their implementation. In a national multicentre survey covering ICUs in China, the incidence of microaspiration was 43.8% (Miao et al. 2023), suggesting that microaspiration remains a clinically relevant problem in routine ICU care. Despite the development of VAP prevention bundles and guidelines, the implementation of nursing practices specifically targeting microaspiration remains insufficiently understood. Recommended core measures for microaspiration prevention, such as standardised oral care and continuous cuff‐pressure monitoring, remain inconsistently implemented in clinical practice, with reported implementation rates ranging from 51.3% to 84.9% (Alanazi and Alonazi 2024; Alhamad and Elsayed 2024; Barnawi et al. 2025). Understanding this evidence‐to‐practice gap is particularly relevant to ICU nurses. A multicenter survey of 626 ICU nurses from 14 tertiary hospitals in Guangxi examined microaspiration assessment in patients with artificial airways. Although 91.7% of the nurses believed that microaspiration assessment should receive the same attention as other ICU complications, only 66.0% reported having actively assessed its occurrence (Zou et al. 2024). However, the study primarily focused on assessment practices and perceived barriers to assessment rather than adherence to a broader set of microaspiration‐prevention practices.

## 2 Background

Previous studies have examined factors relevant to microaspiration assessment and prevention, including nurses' knowledge and skills, work environment, social support, equipment availability and intervention delivery (Kang and Zhou 2024; Miao et al. 2023; Thapa et al. 2024; Zou et al. 2024). However, their research questions and study designs differed. Zou et al. (2024) conducted a multicentre cross‐sectional survey of 626 ICU nurses from 14 tertiary hospitals in Guangxi, focusing on nurses' assessment of microaspiration in patients with artificial airways and perceived barriers to such assessment. Kang and Zhou (2024), in contrast, conducted a single‐hospital clinical intervention study involving 80 patients receiving artificial airway treatment. Their study applied the SHEL model to structure preventive interventions across software, hardware, environmental, and liveware components and evaluated patient‐level clinical outcomes, including aspiration and ventilator‐associated pneumonia. Accordingly, it did not assess ICU nurses' implementation determinants through a survey. Overall, previous research has placed greater emphasis on assessment practices, clinical interventions, and system‐ and context‐related influences than on individual behavioural and psychological determinants among Chinese ICU nurses. To address this gap, the present multicentre cross‐sectional study assessed self‐reported adherence to 22 microaspiration‐prevention practices and 43 implementation‐related items among 476 ICU nurses from 31 ICUs in 17 hospitals in Henan Province. It focused specifically on knowledge, beliefs, intentions, emotions, professional role perceptions, and behavioural regulation, with the Theoretical Domains Framework (TDF) used retrospectively as an interpretive framework. The 43 implementation‐related items were developed independently using a DIBQ‐ and TDF‐informed approach and were not derived from the assessment‐barrier questionnaire used by Zou et al. (2024) or from the SHEL intervention protocol evaluated by Kang and Zhou (2024). The present study was independent of Zou et al. (2024), with no overlap in authors, participating hospitals, participants, questionnaire response data, or datasets. The Kang and Zhou (2024) study was likewise independent, with no overlap in authors, hospital, participants, or dataset. The TDF provides a comprehensive approach to examining individual‐level behavioural and psychological determinants (McGowan et al. 2020) and may help identify potential targets for interventions aimed at strengthening the implementation of microaspiration‐prevention practices.

Accordingly, this study aimed to assess ICU nurses' self‐reported adherence to recommended microaspiration‐prevention practices and to explore potential implementation determinants, using the TDF retrospectively as an interpretive framework.

## 3 Methods

### 3.1 Design

This study used a multicentre, quantitative cross‐sectional design to explore potential determinants of ICU nurses' implementation of microaspiration‐prevention practices. The study was reported in accordance with the Strengthening the Reporting of Observational Studies in Epidemiology (STROBE) guidelines for cross‐sectional studies.

### 3.2 Setting and Participants

The study was conducted in 17 hospitals across four major cities in Henan Province, China, encompassing 31 adult ICUs. General, emergency, cardiac, and specialised ICUs with bed capacities ranging from 3 to 30 beds were considered eligible. Using Kendall's method (Wu 2003), the recommended sample size for a questionnaire survey was 5–10 times the number of items in the questionnaire. The questionnaire comprised 75 items; to account for invalid responses or non‐cooperation, the sample size was increased by 20% over the original estimate, yielding a final sample range of 450–900 respondents. This original sample‐size calculation was based solely on the questionnaire‐item rule and the 20% allowance; it did not specifically account for the three‐level analysis or the number of ICUs contributing observations for the ICU‐level bed‐capacity exposure.

This study used convenience sampling to recruit participants. The research team first contacted the head nurses of the ICUs in each hospital to confirm their willingness to facilitate the study. After the head nurses agreed to facilitate recruitment, ICU nurses from the relevant departments were invited to participate. The inclusion criteria were a valid registered nurse license, at least 1 year of continuous experience in an adult ICU before study initiation, and provision of informed consent after receiving information about the study purpose. Nurses employed part‐time or on leave during the study period were excluded because they were not actively engaged in clinical practice.

### 3.3 Instruments

A self‐administered questionnaire was developed from previous literature and adapted to the context of microaspiration‐prevention practices among clinical nurses in China. The questionnaire comprised 75 items in three sections: nurses' demographic characteristics and professional backgrounds, current microaspiration‐prevention practices in ICUs, and potential implementation determinants. The TDF was used retrospectively as an interpretive framework (Michie 2005).

The first section collected sociodemographic, work‐related, institutional, and training‐related information, including sex, age, hospital level, ICU department, bed capacity, educational level, years of ICU working experience, years working as an ICU specialist nurse, participation in evidence‐based practice training, and participation in microaspiration prevention training.

The second section assessed adherence to microaspiration prevention practices based on current literature and relevant clinical recommendations (Supporting Information 1). To strengthen the clinical and scientific basis of the adherence items, we also mapped the items to intervention‐specific evidence for measures intended to reduce microaspiration and/or VAP, including oral care, subglottic secretion drainage, routine cuff‐pressure monitoring and semirecumbent positioning. These intervention‐specific references were considered together with previous questionnaires, guidelines, and nursing studies to establish the clinical rationale for the adherence items. Some microaspiration‐prevention practice items were adapted from previously published questionnaires and nursing studies rather than reproduced verbatim, and the specific item‐level sources are presented in Supporting Information 1. The strength and specificity of the supporting evidence varied across items. Some practices were directly supported by clinical recommendations, whereas others were condition‐specific, primarily supported as components of multicomponent prevention bundles or by indirect evidence, or supported by mixed or limited evidence when considered as stand‐alone interventions. Accordingly, evidence supporting a multicomponent bundle was not interpreted as demonstrating the independent effectiveness of each individual component. The tool comprised five microaspiration‐prevention domains: basic care, cuff management, subglottic suctioning, enteral nutrition, and risk management, with a total of 22 items (Supporting Information 1). Each item was rated using a five‐point Likert scale (1 = never, 2 = rarely, 3 = sometimes, 4 = often, 5 = always). The original questionnaire did not include a separate ‘not applicable’ response option, and all 22 items were included in the total score. Item‐level information on individual nurses' opportunities to perform each practice was not collected. The total score for this section ranged from 22 to 110 points, with higher scores indicating more frequent self‐reported implementation of the included microaspiration‐prevention practices under the respondent's actual clinical conditions.

The third section contained 43 items intended to explore potential determinants of microaspiration‐prevention implementation (Table 5). Item selection was informed by the Determinants of Implementation Behaviour Questionnaire (DIBQ), which is grounded in the TDF, and by a review of relevant literature (Supporting Information 1). During interpretation, individual items were retrospectively considered in relation to potentially relevant TDF domains based on their content and previous literature. Accordingly, the TDF domain interpretations were exploratory. Items were scored using a five‐point Likert scale ranging from 1 (strongly disagree) to 5 (strongly agree), with higher scores indicating greater endorsement of the corresponding implementation‐related statement. The questionnaire and participant‐level data used in the present study were developed and collected independently. The studies by Zou et al. (2024) and Kang and Zhou (2024) served only as relevant literature sources; there was no sharing of questionnaire response data, participant‐level data, or datasets between the present study and either of those studies.

The DIBQ (Huijg et al. 2014) is based on the TDF and was developed to assess factors influencing healthcare professionals' implementation behaviours. The final DIBQ comprised 93 items across 18 domains, explaining 63.3% of the variance, with internal consistency reliability values ranging from 0.68 to 0.93. The DIBQ assesses key factors influencing healthcare professionals' behaviours and practice implementation, including knowledge and skills, beliefs about capabilities, optimism, and goals as well as external factors such as the socio‐political context and organisation. It also considers patient characteristics, innovation strategy, social influences, and both positive and negative emotions. It additionally addresses behavioural regulation and the nature of the target behaviours, supporting assessment of implementation determinants in healthcare settings.

Five experts with at least 10 years of clinical experience each reviewed the questionnaire content. Two were university professors specialising in critical care nursing, and three were ICU respiratory care specialists. The adherence and implementation‐related sections had scale‐level content validity indices of 0.91 and 0.94, respectively. Preliminary internal consistency was assessed in a separately recruited group of 30 ICU nurses who were not included among the 510 main‐survey respondents or the final analytic sample of 476 nurses. Cronbach's *α* values were 0.934 for the adherence section and 0.846 for the implementation‐related section. These findings provided preliminary evidence of content validity and internal consistency. Comprehensive psychometric validation was not undertaken.

### 3.4 Data Collection

The survey was administered from December 2024 to February 2025 using the Wenjuanxing platform (https://www.wjx.cn), a widely used online survey instrument in China. We initially contacted 17 hospitals in four cities within Henan Province via professional networks. The corresponding author then contacted the head nurses of the ICUs at these institutions to confirm eligibility for participation and provided them with the link to the online survey. At the beginning of the survey, participants were required to read and agree to the consent form, which outlined the study's purpose and provided assurances of anonymity, voluntary participation and confidentiality. Participants could proceed only after providing consent by checking the relevant box. All questionnaire items were mandatory on the online survey platform; therefore, submitted questionnaires had no missing item‐level responses.

### 3.5 Ethical Considerations

This study received approval from the Ethics Committee of Henan Provincial People's Hospital as part of the 2024 Evidence‐Based Nursing Practice Project approval process (Approval/Reference No. HNSYXZKT202423; approval date: July 30, 2024). The nurses were informed that they could withdraw from the survey at any time without any adverse consequences.

### 3.6 Data Analysis

Data were analysed using SPSS version 20.0 (IBM Corp., Armonk, NY, USA). Questionnaires showing identical responses across all items were considered invalid and excluded from the final analysis. The 476 valid questionnaires contained no missing data; therefore, no missing‐data imputation was required. The Kolmogorov–Smirnov test was used to assess the normality of continuous variables. Normally distributed continuous variables were summarised as the mean and standard deviation (SD), whereas non‐normally distributed variables were summarised as the median and interquartile range (IQR). Categorical variables were summarised using frequencies and percentages.

Because adherence scores were not normally distributed, non‐parametric tests were used for bivariate analyses. The Mann–Whitney *U* test was used for two‐group comparisons, and the Kruskal–Wallis *H* test was used for comparisons among three or more groups. The primary multivariable analysis used a three‐level random‐intercept linear mixed‐effects model to account for nurses (Level 1) clustered within 31 ICUs (Level 2) and 17 hospitals (Level 3). The model included random intercepts for hospitals and ICUs nested within hospitals and was estimated using restricted maximum likelihood. The primary multivariable model was adjusted for ICU bed capacity, age, ICU type, educational level, years of ICU working experience, hospital level, evidence‐based practice training, and microaspiration prevention training. Covariates were selected based on clinical and contextual relevance rather than solely on bivariate statistical significance or modifiability. Categorical covariates were entered using indicator coding. As an ICU‐level exposure, ICU bed capacity was modelled as a five‐category predictor (< 5, 5–9, 10–14, 15–19 and ≥ 20 beds); the ≥ 20‐bed category comprised ICUs with capacities ranging from 20 to 30 beds. Bed capacity was not entered as an ordinal score, and no linear trend across categories was assumed. ICU type was represented by four categories: general ICU, medical/cardiopulmonary specialty ICU, surgical/neurosurgical ICU, and emergency/other adult ICU. The reference categories were < 5 beds for ICU bed capacity, general ICU for ICU type, junior college or below for educational level, secondary hospital for hospital level, and no training for both evidence‐based practice training and microaspiration prevention training. Age and ICU working experience categories were assigned representative values in ascending category order (age: 23, 28, 33, 38, 43 and 48 years; ICU working experience: 2, 4, 6, 8, 10, 12, 14, 16, 18 and 20 years) and entered as continuous covariates in the primary model. Adjusted unstandardised coefficients (B), standard errors, 95% confidence intervals, and *p* values were reported, together with a four‐degree‐of‐freedom overall Wald test for bed capacity.

The 22‐item total adherence score was the dependent variable. Multicollinearity among the fixed‐effect covariates was assessed using variance inflation factors. Sensitivity analyses included generalised estimating equations with the ICU as the clustering unit, a Gaussian distribution with identity link, exchangeable and independent working correlation structures and cluster‐robust standard errors. To assess the robustness of inference with 31 ICU clusters, a finite‐cluster reference‐distribution sensitivity analysis used *t*(30) reference distributions for individual coefficients and *F*(4,30) for the overall bed‐capacity test. For the applicability‐focused sensitivity analysis, an additional outcome was calculated after excluding all eight items explicitly identified as condition‐specific in Supporting Information 1: Items 7, 11, 12, 13, 14, 15, 16 and 22. The mean of the remaining 14 items was calculated to retain the original 1–5 response metric, and the fully adjusted three‐level mixed‐effects model and ICU‐clustered generalised estimating equation analyses were repeated using this outcome. Because item‐level applicability was not collected for individual participants, this analysis was considered a robustness analysis rather than a definitive applicability‐adjusted scoring method and did not replace the 22‐item outcome used in the primary analysis. Sensitivity analyses used coarser categorical specifications of age and ICU working experience or orthogonalised ICU working experience with respect to age, and some models omitted age or ICU working experience. Maximum likelihood was used only for comparing random‐effects structures. Regression findings were interpreted as exploratory associations rather than as evidence of causality.

For the primary 22‐item three‐level mixed‐effects model, conditional residuals (observed values minus fitted values including the random effects) were assessed using a normal Q–Q plot, skewness, kurtosis, the Jarque–Bera test, and a residual‐versus‐fitted‐value plot. Cluster‐level influence was evaluated by refitting the model after omitting one hospital or one ICU at a time. For each refitted model, convergence status and changes in the direction and statistical significance of five core coefficients were recorded: the 10–14‐bed, 15–19‐bed and ≥ 20‐bed contrasts relative to < 5 beds, and the evidence‐based practice training and microaspiration‐prevention training coefficients. Cook's distance and the Breusch–Pagan test were calculated from an auxiliary ordinary least‐squares model and were not treated as formal diagnostics of the three‐level mixed‐effects model. No observations were excluded solely on the basis of the auxiliary Cook's distance threshold.

Total and domain adherence scores were expressed as percentages of their respective maximum possible scores and summarised descriptively. A two‐sided *p* value < 0.05 was considered statistically significant.

## 4 Results

### 4.1 Characteristics of Study Participants

A total of 510 ICU nurses completed the survey. Of these, 34 questionnaires with identical responses across all items were excluded as invalid, leaving 476 valid questionnaires for the final analysis. No data were missing for any of the variables included in the analysis. The 31 ICUs were distributed across the five bed‐capacity categories as follows: < 5 beds, 6 ICUs (85 nurses); 5–9 beds, 6 ICUs (116 nurses); 10–14 beds, 6 ICUs (96 nurses); 15–19 beds, 7 ICUs (99 nurses); and ≥ 20 beds, 6 ICUs (80 nurses). Table 1 summarises the demographic and clinical characteristics of the 476 participants. Most participants were female nurses (75%). Most participants were aged 21–35 years, with 21.43% aged 21–25 years and 24.79% aged 31–35 years. At the hospital level, 50.21% of the participants were employed in secondary hospitals and 49.79% in tertiary hospitals. The largest ICU subgroups were the general ICU (16.18%) and respiratory ICU (13.66%). Regarding educational attainment, the largest subgroup (37.61%) held a bachelor's degree, and 50.42% had 1–3 years of professional experience as ICU specialist nurses. In addition, 69.54% had received training in evidence‐based practice, and 71.22% had received training in microaspiration prevention.

**TABLE 1:** Demographic and clinical characteristics of participants (*N* = 476).

Variables	Categories	Number	Percentage (%)	Cumulative percentage (%)
Sex	Male	119	25	25
	Female	357	75	100
Age	21–25	102	21.43	21.43
	26–30	113	23.74	45.17
	31–35	118	24.79	69.96
	36–40	37	7.77	77.73
	41–45	45	9.45	87.18
	46–50	61	12.82	100
Hospital level	Secondary	239	50.21	50.21
	Tertiary	237	49.79	100
ICU departments	General ICU	77	16.18	16.18
	RICU (respiratory ICU)	65	13.66	29.83
	Cardiac ICU (CCU)	44	9.24	39.08
	Surgical ICU (SICU)	49	10.29	49.37
	Neurosurgery ICU (NICU)	34	7.14	56.51
	Medicine ICU (MICU)	44	9.24	65.76
	Cardiac surgery ICU (CSICU)	28	5.88	71.64
	EICU (emergency ICU)	35	7.35	78.99
	NICU (neurology ICU)	37	7.77	86.76
	KICU (kidney ICU)	38	7.98	94.75
	Other adult ICU	25	5.25	100
Bed capacity	< 5 beds	85	17.86	17.86
	5–9 beds	116	24.37	42.23
	10–14 beds	96	20.17	62.39
	15–19 beds	99	20.8	83.19
	≥ 20 beds	80	16.81	100
Educational level	A. Junior college and below	90	18.91	18.91
	B. Bachelor's degree	179	37.61	56.51
	C. Master's degree	164	34.45	90.97
	D. Doctorate	43	9.03	100
Years of ICU working experience	A. > 1, ≤ 3 years	171	35.92	35.92
	B. > 3, ≤ 5 years	92	19.33	55.25
	C. > 5, ≤ 7 years	70	14.71	69.96
	D. > 7, ≤ 9 years	20	4.2	74.16
	E. > 9, ≤ 11 years	18	3.78	77.94
	F. > 11, ≤ 13 years	22	4.62	82.56
	G. > 13, ≤ 15 years	31	6.51	89.08
	H. > 15, ≤ 17 years	20	4.2	93.28
	I. > 17, ≤ 19 years	16	3.36	96.64
	J. > 19 years	16	3.36	100
Years of working as an ICU specialist nurse	A. > 1, ≤ 3 years	240	50.42	50.42
	B. > 3, ≤ 5 years	71	14.92	65.34
	C. > 5, ≤ 7 years	40	8.4	73.74
	D. > 7, ≤ 9 years	29	6.09	79.83
	E. > 9, ≤ 11 years	10	2.1	81.93
	F. > 11, ≤ 13 years	31	6.51	88.45
	G. > 13, ≤ 15 years	21	4.41	92.86
	H. > 15, ≤ 17 years	18	3.78	96.64
	I. > 17, ≤ 19 years	16	3.36	100
	J. > 19 years	0	0	100
Participation in evidence‐based practice training	A. No	145	30.46	30.46
	B. Yes	331	69.54	100
Participation in microaspiration prevention training	A. No	137	28.78	28.78
	B. Yes	339	71.22	100

*Note:* Years of specialised nursing experience*: Nurses who participated in ICU specialist nurse training, passed the assessment, and obtained certification as ICU specialist nurses. The ≥ 20‐bed category included ICUs with bed capacities ranging from 20 to 30 beds. For the bed‐capacity categories, the numbers of contributing ICUs were 6, 6, 6, 7 and 6 for < 5, 5–9, 10–14, 15–19 and ≥ 20 beds, respectively; the corresponding numbers of nurses were 85, 116, 96, 99 and 80.

### 4.2 ICU Nurses' Adherence to Microaspiration Prevention Practices

The mean total adherence score was 77.17 (SD = 18.42; total score range: 22–110), corresponding to 70.15% of the maximum possible score. Table 2 shows that risk management had the lowest item‐level mean score among the five domains (3.36 ± 1.24; range: 1–5; percentage of the maximum possible score: 67.2%), whereas basic care had the highest item‐level mean score (3.68 ± 1.17; range: 1–5; percentage of the maximum possible score: 73.6%). At the item‐level (Supporting Information 2), ‘Perform oral care for the patient at least twice daily’ (3.70 ± 1.37) was the highest‐scoring item, followed by ‘Use a combined oral care approach involving tooth brushing, rinsing, and suctioning’ (3.68 ± 1.34) and ‘Elevate the head of bed to 30°–45° for intubated patients to reduce the risk of microaspiration’ (3.68 ± 1.35). The lowest‐scoring items included ‘Perform continuous risk assessment for microaspiration in high‐risk patients during nursing care’ (3.33 ± 1.46), ‘Have the ability to use or assist in using bedside gastric ultrasound to assess the patient's gastric residual volume (GRV), if necessary’ (3.35 ± 1.45), and ‘Have the ability to identify patients at high‐risk of microaspiration’ (3.37 ± 1.46).

**TABLE 2:** Mean and percentage scores of adherence to the microaspiration prevention (*N* = 476).

Dimension	Possible total score range	Mean (SD)	Percentage score (%)
Basic care	4–20	3.68 (1.17)^a^	73.6
Cuff management	6–30	3.50 (1.10)^a^	70.0
Subglottic suctioning	3–15	3.44 (1.17)^a^	68.8
Enteral nutrition	5–25	3.54 (1.11)^a^	70.8
Risk management	4–20	3.36 (1.24)^a^	67.20
Total score—Section 2	22–110	77.17 (18.42)^b^	70.15

*Note:* Percentage score was calculated as the observed mean score divided by the maximum possible score and multiplied by 100. For domain rows, the item‐level mean score was divided by 5; for the total score row, the total mean score was divided by 110.

^a^ Mean score per item (SD).

^b^ Total score, mean (SD).

### 4.3 Factors Associated With Adherence to Microaspiration Prevention

In bivariate analyses (Table 3), age, ICU department type, unit bed capacity, educational level, years of ICU working experience, years working as an ICU specialist nurse, evidence‐based practice training, and microaspiration‐prevention training were significantly associated with the overall adherence score. The Kruskal–Wallis test indicated significant differences among the 11 departments (*H* = 51.993, *p* < 0.001). Across departments, median (Mdn) total adherence scores ranged from 70.0 to 89.0 points (full range of 19.0 points); the general ICU (Mdn = 89.0) and respiratory ICU (Mdn = 89.0) had the highest scores, whereas the emergency ICU had the lowest score at 70.0. Adherence differed significantly across bed‐capacity groups (*H* = 30.58, *p* < 0.001). ICUs with < 5 beds and those with 5–9 beds had the highest median adherence score (Mdn = 88.0 in both groups), whereas ICUs with 15–19 beds had the lowest median score (Mdn = 73.0). Nurses who had received evidence‐based practice training had higher adherence scores than those who had not (Mdn = 87.0 vs. 75.0; *U* = 18,218.5, *p* < 0.001). Similarly, nurses who had received microaspiration‐prevention training had higher adherence scores than those who had not (Mdn = 86.0 vs. 74.0; *U* = 18,729.5, *p* < 0.001).

**TABLE 3:** Bivariate analysis of factors associated with adherence to the microaspiration prevention (*N* = 476).

Variable category	Group/subgroup	Sample size (*n*)	Median (Mdn)	Interquartile range (P25, P75)	Test statistic	*p*
ICU department	General ICU	77	89	(84.0, 94.0)	*H* ^a^ = 51.993	< 0.001
	Respiratory ICU (RICU)	65	89	(83.0, 93.0)		
	Cardiac ICU (CCU)	44	85.5	(61.3, 88.0)		
	Surgical ICU (SICU)	49	86	(54.0, 90.5)		
	Neurosurgery ICU	34	85.5	(65.0, 93.0)		
	Medical ICU (MICU)	44	77.5	(55.3, 91.0)		
	Cardiac surgery ICU (CSICU)	28	75.5	(47.5, 87.8)		
	Emergency ICU (EICU)	35	70	(55.0, 86.0)		
	Neurology ICU (NICU)	37	75	(53.0, 90.0)		
	Kidney ICU (KICU)	38	76	(52.5, 88.3)		
	Other adult ICU	25	72	(51.0, 86.5)		
Bed capacity	< 5 beds	85	88	(80.0, 92.0)	*H* ^a^ = 30.579	< 0.001
	5–9 beds	116	88	(75.0, 92.8)		
	10–14 beds	96	82	(53.3, 90.0)		
	15–19 beds	99	73	(51.0, 90.0)		
	≥ 20 beds	80	74.5	(58.0, 88.0)		
Training	Evidence‐based practice training	—	—	—	*U* ^b^ = 18,218.5	< 0.001
	Untrained	145	75	(55.5, 88.0)		
	Trained	331	87	(67.0, 92.0)		
	Microaspiration prevention training	—	—	—	*U* ^b^ = 18,729.5	< 0.001
	Untrained	137	74	(57.0, 89.0)		
	Trained	339	86	(67.0, 92.0)		
Age	21–25 years	102	86.5	(73.8, 92.0)	*H* ^a^ = 32.067	< 0.001
	26–30 years	113	88	(70.0, 93.0)		
	31–35 years	118	87	(65.5, 92.0)		
	36–40 years	37	74	(61.5, 87.0)		
	41–45 years	45	68	(51.0, 89.0)		
	46–50 years	61	68	(47.5, 86.5)		
Sex	Male	119	85	(66.0, 91.0)	*U* ^b^ = 20,561.00	0.600
	Female	357	85	(62.0, 91.0)		
Hospital level	Secondary	239	84	(62.0, 91.0)	*U* ^b^ = 27,602.500	0.632
	Tertiary	237	86	(63.0, 91.0)		
Educational level	Associate degree or below	90	77	(54.3, 90.0)	*H* ^a^ = 30.920	< 0.001
	Bachelor's degree	179	75	(52.0, 90.0)		
	Master's degree	164	87.5	(78.0, 92.0)		
	Doctoral degree	43	88	(74.0, 93.0)		
Years of ICU working experience	> 1, ≤ 3	171	87	(72.0, 92.0)	*H* ^a^ = 38.007	< 0.001
	> 3, ≤ 5	92	87	(64.5, 91.0)		
	> 5, ≤ 7	70	87	(69.8, 92.0)		
	> 7, ≤ 9	20	66	(51.0, 84.3)		
	> 9, ≤ 11	18	66.5	(57.8, 89.0)		
	> 11, ≤ 13	22	84	(54.0, 91.3)		
	> 13, ≤ 15	31	68	(46.0, 78.0)		
	> 15, ≤ 17	20	80.5	(58.5, 89.3)		
	> 17, ≤ 19	16	73	(50.8, 89.0)		
	> 19	16	64.5	(47.0, 87.5)		
Years of working as an ICU specialist nurse	> 1, ≤ 3	240	87	(69.3, 92.0)	*H* ^a^ = 23.087	0.003
	> 3, ≤ 5	71	86	(66.0, 90.0)		
	> 5, ≤ 7	40	79.5	(52.0, 89.8)		
	> 7, ≤ 9	29	83	(63.0, 89.0)		
	> 9, ≤ 11	10	77	(53.3, 91.5)		
	> 11, ≤ 13	31	74	(52.0, 87.0)		
	> 13, ≤ 15	21	83.000	(47.5, 92.0)		
	> 15, ≤ 17	18	70.500	(46.5, 91.5)		
	> 17, ≤ 19	16	57.500	(48.0, 80.5)		

*Note: H* ^a^, Kruskal‐Wallis *H* test; *U* ^b^, Mann–Whitney *U* test.

In the fully adjusted three‐level mixed‐effects model, ICU bed capacity showed an overall association with adherence (Wald *χ* ^2^(4) = 13.234, *p* = 0.010). Compared with ICUs with < 5 beds, adjusted adherence scores were lower for ICUs with 10–14 beds (*B* = −7.161, 95% CI, −12.648 to −1.674, *p* = 0.011), 15–19 beds (*B* = −8.383, 95% CI, −15.275 to −1.491, *p* = 0.017), and ≥ 20 beds (*B* = −7.101, 95% CI, −13.518 to −0.684, *p* = 0.030), whereas the 5–9‐bed group did not differ significantly (*B* = −0.772, 95% CI, −6.249 to 4.705, *p* = 0.782). Evidence‐based practice training (*B* = 3.971, 95% CI, 0.515 to 7.426, *p* = 0.024) and microaspiration‐prevention training (*B* = 3.947, 95% CI, 0.559 to 7.335, *p* = 0.022) were independently associated with higher adherence scores. Among the additional covariates, older age was associated with lower adherence scores (*B* = −0.619, 95% CI, −1.030 to −0.208, *p* = 0.003). Compared with nurses with junior college education or below, those with a master's degree (*B* = 6.335, 95% CI, 1.921 to 10.748, *p* = 0.005) or doctoral degree (*B* = 9.203, 95% CI, 3.095 to 15.312, *p* = 0.003) had higher adjusted adherence scores, whereas bachelor's education was not independently associated with adherence. ICU type, hospital level, and ICU working experience were not independently associated with adherence (Table 4). The hospital random‐intercept variance was 24.762, ICU‐level incremental variance was approximately zero, and residual variance was 260.303; the estimated hospital‐level and combined hospital‐plus‐ICU intraclass correlation coefficients were both 8.69%. Table 4 presents all fully adjusted coefficients. In the applicability‐focused sensitivity analysis excluding all eight items identified as condition‐specific in Supporting Information 1, the 14‐item mixed‐effects model retained lower implementation‐frequency scores for ICUs with 10–14, 15–19, and ≥ 20 beds than for those with < 5 beds, and evidence‐based practice training remained positively associated with the outcome. The coefficient for microaspiration‐prevention training remained positive but was no longer statistically significant. Corresponding ICU‐clustered generalised estimating equation analyses showed similar directions, although statistical significance varied across specifications (Supporting Information 3).

**TABLE 4:** Fully adjusted three‐level mixed‐effects model of ICU nurses' adherence to microaspiration prevention (*N* = 476).

Variables	*B*	SE	95% CI	*p*
Constant	91.330	6.762	78.076 to 104.583	< 0.001
Bed capacity: 5–9 beds versus < 5 beds	−0.772	2.794	−6.249 to 4.705	0.782
Bed capacity: 10–14 beds versus < 5 beds	−7.161	2.800	−12.648 to −1.674	0.011
Bed capacity: 15–19 beds versus < 5 beds	−8.383	3.516	−15.275 to −1.491	0.017
Bed capacity: ≥ 20 beds versus < 5 beds	−7.101	3.274	−13.518 to −0.684	0.030
ICU type: medical/cardiopulmonary specialty versus general ICU	3.949	3.420	−2.755 to 10.653	0.248
ICU type: surgical/neurosurgical versus general ICU	−1.870	3.189	−8.121 to 4.381	0.558
ICU type: emergency/other adult ICU versus general ICU	−0.074	3.345	−6.631 to 6.482	0.982
Education: bachelor's degree versus junior college or below	−1.733	2.209	−6.063 to 2.596	0.433
Education: master's degree versus junior college or below	6.335	2.252	1.921 to 10.748	0.005
Education: doctoral degree versus junior college or below	9.203	3.117	3.095 to 15.312	0.003
Hospital level: tertiary versus secondary	0.921	2.960	−4.881 to 6.723	0.756
Age representative value (per year)	−0.619	0.210	−1.030 to −0.208	0.003
ICU working‐experience representative value (per year)	0.312	0.326	−0.327 to 0.952	0.338
Evidence‐based practice training: yes versus no	3.971	1.763	0.515 to 7.426	0.024
Microaspiration‐prevention training: yes versus no	3.947	1.729	0.559 to 7.335	0.022

*Note:* Bed capacity overall: Wald *χ* ^2^(4) = 13.234, *p* = 0.010. The dependent variable was the 22‐item total adherence score. The model included random intercepts for hospitals and for ICUs nested within hospitals and was estimated using restricted maximum likelihood. Fixed effects included bed capacity, age, ICU type, educational level, ICU working experience, hospital level, evidence‐based practice training, and microaspiration‐prevention training. Bed capacity was entered categorically with < 5 beds as the reference; the ≥ 20‐bed category included ICUs with 20–30 beds. ICU type reference = general ICU; education reference = junior college or below; hospital level reference = secondary; training reference = no. Hospital random‐intercept variance = 24.762; ICU‐within‐hospital random‐intercept variance = 0.000004; residual variance = 260.303; hospital‐level ICC = 8.69%; combined hospital‐plus‐ICU ICC = 8.69%.

Diagnostic assessment of the primary model identified some departures from standard residual assumptions. Conditional residuals showed slight left skewness (skewness = −0.43; kurtosis = 2.60), and the Jarque–Bera test indicated departure from normality (*χ* ^2^ = 17.76, *p* < 0.001; Figure S1). The residual‐versus‐fitted‐value plot showed visible structure and unequal residual variance (Figure S2). In an auxiliary ordinary least‐squares model, the Breusch–Pagan statistic was *χ* ^2^ = 35.46 (*p* = 0.002); this result was treated as auxiliary information and not as a formal diagnostic of the three‐level mixed‐effects model. Leave‐one‐cluster‐out analyses were also performed. At the hospital level, models omitting H10 and H15 did not meet the convergence criterion; at the ICU‐level, models omitting I12 and I24 did not meet the convergence criterion. Among the converged refitted models, the directions of the five core coefficients were unchanged, although statistical significance varied in some specifications. Nonconverged refitted models were not interpreted as reliable evidence. Cook's distance screening did not lead to automatic exclusion of any observations. Complete hospital‐ and ICU‐level leave‐one‐cluster‐out results, including convergence status, are reported in Supporting Information 3 (Tables S4 and S5).

### 4.4 Descriptive Assessment of Potential Microaspiration‐Prevention Implementation Determinants

Table 5 summarises the descriptive scores for the 43 implementation‐related items. The three lowest‐scoring items were ‘I intend to deliver microaspiration prevention following the implementation recommendations in the next three months’ (3.10 ± 1.72), ‘I usually prioritise the implementation of microaspiration‐prevention measures over other routine tasks’ (3.21 ± 1.68), and ‘Delivering microaspiration prevention following the implementation recommendations is part of my work as a nurse’ (3.29 ± 1.48). The three highest‐scoring items were ‘Microaspiration prevention costs little time to deliver’ (4.19 ± 1.05), ‘Microaspiration prevention is compatible with daily practice’ (4.15 ± 1.08), and ‘For me, delivering microaspiration prevention following the implementation recommendations is interesting’ (3.94 ± 1.29). These scores highlight relatively lower‐ and higher‐scoring implementation‐related areas for further investigation.

**TABLE 5:** Descriptive scores for implementation‐related items concerning microaspiration prevention (*N* = 476).

Item	Domain	Mean	Sd.	Percentage score (%)
I intend to deliver microaspiration prevention following the implementation recommendations in the next 3 months.	Intentions	3.1	1.72	62.00
I usually prioritise the implementation of microaspiration prevention measures over other routine tasks.	Goals	3.21	1.68	64.20
Delivering microaspiration prevention following the implementation recommendations is part of my work as a nurse.	Social/professional role and identity	3.29	1.48	65.80
I can count on support from the management of the organisation I work in when things get tough around delivering microaspiration prevention following the implementation recommendations.	Organisation	3.36	1.48	67.20
Professionals with whom I deliver microaspiration prevention are helpful with delivering microaspiration prevention following the implementation recommendations.	Social influences	3.36	1.44	67.20
The management of the organisation I work in is helpful with delivering microaspiration prevention following the implementation recommendations.	Organisation	3.39	1.5	67.80
Objectives of microaspiration prevention and my role in this are clearly defined for me.	Knowledge	3.43	1.4	68.60
Most people who are important to me think that I should deliver microaspiration prevention following the implementation recommendations.	Social influences	3.43	1.52	68.60
Hospitals organises intervision meetings for professionals.	Innovation strategy	3.44	1.34	68.80
Hospital provides insights into results of microaspiration prevention.	Innovation strategy	3.44	1.53	68.80
I am confident that I can deliver microaspiration prevention following the implementation recommendations even when other professionals with whom I deliver microaspiration prevention do not do this.	Beliefs about capabilities	3.45	1.53	69.00
The management of the organisation I work in is willing to listen to my problems with delivering microaspiration prevention following the implementation recommendations.	Organisation	3.45	1.5	69.00
In the organisation I work, all necessary resources are available to deliver microaspiration prevention.	Organisation	3.47	1.52	69.40
I am confident that I can deliver microaspiration prevention following the implementation recommendations even when there is little time.	Beliefs about capabilities	3.57	1.32	71.40
When I work with microaspiration prevention, I feel calm.	Positive emotions	3.57	1.4	71.40
Delivering microaspiration prevention following the implementation recommendations is something I do without having to consciously remember.	Nature of the behaviours	3.58	1.32	71.60
When I work with microaspiration prevention, I feel agitated.	Negative emotions	3.58	1.42	71.60
I am confident that I can deliver microaspiration prevention following the implementation recommendations even when participants are not motivated.	Beliefs about capabilities	3.61	1.28	72.20
When I work with microaspiration prevention, I feel relaxed.	Positive emotions	3.62	1.39	72.40
When I work with microaspiration prevention, I feel nervous.	Negative emotions	3.63	1.45	72.60
I am practiced to deliver microaspiration prevention following the implementation recommendations.	Skills	3.63	1.31	72.60
For me, performing the evaluation is (very difficult – very easy).	Beliefs about capabilities	3.65	1.31	73.00
I am confident that I can deliver microaspiration prevention intervention following the implementation recommendations.	Beliefs about capabilities	3.66	1.35	73.20
In my work as a nurse, in uncertain times, I usually expect the best.	Optimism	3.68	1.33	73.60
In my work as a nurse, overall, I expect more good things to happen than bad.	Optimism	3.69	1.26	73.80
I have the skills to deliver microaspiration prevention following the implementation recommendations.	Skills	3.69	1.29	73.80
For me, delivering microaspiration prevention following the implementation recommendations is (very difficult – very easy).	Beliefs about capabilities	3.77	1.42	75.40
If I deliver microaspiration prevention following the implementation recommendations, this will strengthen the collaboration with professionals with whom I deliver microaspiration prevention.	Beliefs about consequences	3.79	1.32	75.80
Delivering microaspiration prevention following the implementation recommendations is something I do automatically.	Nature of the behaviours	3.8	1.36	76.00
Professionals with whom I deliver microaspiration prevention think I should deliver microaspiration prevention following the implementation recommendations.	Social influences	3.82	1.32	76.40
When I work with microaspiration prevention I feel comfortable.	Positive emotions	3.84	1.25	76.80
I know how to deliver microaspiration prevention following the implementation recommendations.	Knowledge	3.86	1.12	77.20
I have control over delivering microaspiration prevention following the implementation recommendations.	Beliefs about capabilities	3.86	1.35	77.20
When I deliver microaspiration prevention following the implementation recommendations, I get recognition from the work context.	Beliefs about consequences	3.87	1.31	77.40
For me, delivering microaspiration prevention following the implementation recommendations is (not worthwhile at all – very worthwhile).	Beliefs about consequences	3.87	1.34	77.40
If I deliver microaspiration prevention following the implementation recommendations, microaspiration prevention will be most effective.	Beliefs about consequences	3.87	1.28	77.40
I have been trained in delivering microaspiration prevention following the implementation recommendations.	Skills	3.91	1.32	78.20
With regard to microaspiration prevention, I know what my responsibilities are.	Knowledge	3.91	1.26	78.20
I have a clear plan under what circumstances I will deliver microaspiration prevention following the implementation recommendations.	Behavioural regulation	3.92	1.25	78.40
I have a clear plan of how I will deliver microaspiration prevention following the implementation recommendations.	Behavioural regulation	3.93	1.24	78.60
For me, delivering microaspiration prevention following the implementation recommendations is (not interesting at all – very interesting).	Beliefs about consequences	3.94	1.29	78.80
Microaspiration prevention is compatible with daily practice.	Innovation	4.15	1.08	83.00
Microaspiration prevention costs little time to deliver.	Innovation	4.19	1.05	83.80
Total score—Section 3		157.25	14.54	73.14

*Note:* Percentage score was calculated as the observed mean score divided by the maximum possible score and multiplied by 100. For each item, the item mean score was divided by 5.

## 5 Discussion

This study investigated ICU nurses' self‐reported adherence to microaspiration‐prevention practices and potential implementation determinants. Although a long‐term quality‐improvement study and a systematic review with meta‐analysis have shown that multicomponent ventilator‐associated pneumonia prevention bundles can reduce ventilator‐associated pneumonia incidence and improve related clinical outcomes (Maertens et al. 2025; Martinez‐Reviejo et al. 2023), these studies primarily demonstrate bundle‐level effects on clinical outcomes. Evidence remains limited regarding intensive care nurses' adherence to microaspiration‐specific preventive practices targeting this upstream mechanism of ventilator‐associated pneumonia. Moreover, standardised measures, publicly accessible adherence data, and evidence on potential implementation determinants remain limited. Accordingly, the item‐level findings should be interpreted as patterns of self‐reported adherence to practices included in microaspiration‐prevention care rather than as evidence that each individual practice independently reduces microaspiration or ventilator‐associated pneumonia.

This study identified variation across microaspiration‐prevention domains and individual practices. The adherence scores represent nurses' self‐reported implementation‐frequency of the included practices under their actual clinical conditions. Because the questionnaire did not assess item‐level applicability, the scores may reflect not only individual implementation behaviour but also differences in patient eligibility, treatment modality, equipment availability, local protocols, and nurses' opportunities or authority to perform particular practices. The domain of basic nursing measures had the highest overall adherence score, with adherence to maintaining head‐of‐bed elevation at 30°–45° reaching 73.6% of the maximum possible score. This relatively high score may reflect the extensive evidence supporting these basic nursing measures in reducing microaspiration (Shi et al. 2013; Unahalekhaka et al. 2025). Zhang et al. (2022) found that the incidence of microaspiration in ICUs was 77.11% (192/249), with higher rates observed among patients positioned below 30° and those receiving gastric tube feeding. The higher scores for basic nursing measures may also be related to the high standardisation of these procedures, their frequent implementation, and their inclusion in routine competency assessments. Within the risk‐management domain, the item concerning the use of or assistance with bedside gastric ultrasonography for gastric residual‐volume assessment had a mean score of 3.35 out of 5, corresponding to 67.0% of the maximum possible score (Supporting Information 2). Because this item was explicitly conditional (‘if necessary’) and item‐level applicability was not assessed, the observed score should not be interpreted solely as inadequate adherence. The score may reflect multiple factors: first, critical care ultrasonographic monitoring requires nurses to undergo specialised training and accumulate experience; second, the availability of necessary equipment, such as bedside ultrasonography devices, may vary across clinical settings; and third, exploratory interpretation of the item scores suggested possible uncertainty regarding nursing responsibility for microaspiration prevention, as reflected by the mean score of 3.29 for the relevant item in Table 5 (Lin et al. 2020; Respiratory Therapy Group of the Respiratory Disease Branch of the Chinese Medical Association 2014; SuWen et al. 2025). Furthermore, the items concerning perceived management support and the helpfulness of colleagues both had mean scores of 3.36 (Table 5), suggesting that organisational and social support may warrant further investigation. Collectively, these factors may influence the implementation of complex interventions, and similar challenges have been reported in other studies. Because the symptoms of microaspiration are often atypical (e.g., absence of coughing), clinical identification is challenging, and existing assessment methods can be complex and costly. Specialised critical care skills training (such as bedside ultrasonography) and clearer delineation of nursing responsibilities may therefore help address some of these challenges.

After accounting for clustering of nurses within ICUs and hospitals and adjusting for age, ICU type, education, ICU working experience, hospital level, and the two training exposures, ICU bed capacity remained associated with adherence. Compared with ICUs with < 5 beds, adjusted scores were lower in the 10–14, 15–19, and ≥ 20‐bed groups, whereas the 5–9‐bed group did not differ significantly. This category‐specific pattern should not be interpreted as a simple linear trend across the five bed‐capacity categories. Evidence‐based practice training and microaspiration‐prevention training were both independently associated with higher adherence, broadly consistent with previous research on nurses' adherence to evidence‐based preventive practices (Song et al. 2024). The bed‐capacity contrasts were generally similar in direction across the generalised estimating equation models, finite‐cluster reference‐distribution sensitivity analysis, applicability‐focused 14‐item analyses excluding all eight items identified as condition‐specific in Supporting Information 1, and alternative age/experience specifications, although statistical significance varied across specifications. In the 14‐item analyses, evidence‐based practice training remained positively associated with the outcome, whereas the coefficient for microaspiration‐prevention training remained positive but was not statistically significant.

The lower adherence observed in ICUs with 10–14, 15–19, or ≥ 20 beds may be related to structural features such as more complex workflows, longer communication chains, less direct supervision, and more difficult coordination of resources. Beyond workflow‐related explanations, differences in continuing education opportunities and knowledge reinforcement may also have contributed to this result. Previous studies by Labeau et al. showed persistent knowledge gaps among ICU nurses regarding evidence‐based infection prevention, including VAP prevention, and provided validated instruments for assessing such knowledge (Labeau et al. 2007, 2008, 2009). Although nurses' knowledge was not directly measured in the present study, one possible explanation is that smaller units may be better positioned to organise continuing education, reinforce recommended practices, and provide timely bedside feedback owing to smaller staff numbers and simpler coordination structures. The positive associations between both forms of training and adherence are consistent with training exposure being relevant to adherence, although knowledge itself was not directly assessed.

The bivariate analysis showed that nurses working in respiratory ICUs had the highest adherence scores; however, ICU type was not independently associated with adherence after multivariable adjustment. Age and educational level also showed adjusted associations. Older age was associated with lower adherence, whereas master's and doctoral degrees, but not a bachelor's degree, were associated with higher adherence relative to junior college education or below. Because these variables were included primarily as potential confounders rather than as the study's principal exposures, these secondary findings warrant cautious interpretation and further investigation. The unadjusted departmental pattern may partly reflect differences in case mix, training exposure, unit organisation and other measured covariates. Taken together, the adjusted findings indicate that ICU bed capacity and training exposure were more consistently associated with adherence in this dataset. Nurse managers, particularly in larger ICUs, may consider strengthening structured continuing education, regular knowledge reinforcement, and bedside supervision of microaspiration‐prevention practices to reduce variability in adherence.

From an exploratory TDF perspective, the higher‐ and lower‐scoring items were retrospectively interpreted in relation to potentially relevant TDF domains and constructs based on their content and previous literature. The three highest‐scoring implementation‐related items reflected time efficiency, workflow compatibility and interest. The highest‐scoring item (‘Microaspiration prevention costs little time to deliver’; mean ± SD: 4.19 ± 1.05) was interpreted as aligning primarily with the innovation domain, specifically the innovation characteristics construct. In light of literature emphasising time efficiency (Reese et al. 2022), this high score suggests that perceived time efficiency may be relevant to implementation. The second‐highest‐scoring item (‘Microaspiration prevention is compatible with daily practice’; mean ± SD: 4.15 ± 1.08) was likewise interpreted as relating to the innovation characteristics construct. This item concerns whether the measures are compatible with existing workflows (Haakma et al. 2022), which may affect team and organisational acceptance. The third‐highest‐scoring item (‘For me, delivering microaspiration prevention following the implementation recommendations is interesting’; mean ± SD: 3.94 ± 1.29) was interpreted as relating to the beliefs about consequences domain, specifically the attitude construct. Its relatively high score may indicate that some nurses find implementation interesting or satisfying, which may contribute to intrinsic motivation. However, the high standard deviation (SD = 1.290) points to variability among individuals.

Among the implementation‐related items, the lowest‐scoring item was ‘I intend to deliver microaspiration prevention following the implementation recommendations in the next three months’ (mean ± SD: 3.10 ± 1.72), which was interpreted as relating to the intentions domain. This relatively low score suggests that near‐term implementation intention may represent a challenge warranting further investigation. The second‐lowest‐scoring item (‘I usually prioritise the implementation of microaspiration prevention measures over other routine tasks’; mean ± SD: 3.21 ± 1.68) was interpreted as relating to the goals domain, specifically the priority construct, which may reflect competing priorities between microaspiration prevention measures and other clinical tasks in resource‐constrained settings. The third‐lowest‐scoring item was ‘As a nurse, implementing microaspiration prevention measures is my job’ (mean ± SD: 3.29 ± 1.48). This item was interpreted as relating to the social/professional role and identity domain, specifically the professional role construct, and its relatively low score may indicate uncertainty regarding professional responsibility for microaspiration prevention. The relatively large standard deviation indicates substantial variability in responses among participants. Taken together, these relatively lower‐scoring items may indicate potential implementation challenges related to near‐term intention, competing clinical priorities and professional role clarity. Because no prespecified threshold was used to classify items as barriers or facilitators and the TDF mapping was conducted retrospectively, these findings should be interpreted as descriptive and exploratory.

### 5.1 Implications for Nursing Practice

The findings may inform ICU nursing practice, management and education. For ICU nurses, the lower‐scoring implementation‐related items may prompt reflection on near‐term implementation intentions, competing clinical priorities, and perceptions of professional responsibility. Nurse managers may strengthen structured microaspiration‐prevention and evidence‐based practice training, clarify nursing responsibilities, embed key preventive practices into routine workflows and checklists, and provide regular bedside supervision and feedback. Nurse educators may incorporate scenario‐based and skills‐focused teaching to support role clarity, prioritisation and workflow integration. Because adjusted adherence was lower in the 10–14, 15–19, and ≥ 20‐bed groups than in ICUs with < 5 beds, these three bed‐capacity groups may warrant additional managerial attention. Given the cross‐sectional design, these strategies should be regarded as priorities for further evaluation rather than as established effective interventions.

### 5.2 Limitations

This study has some limitations. First, the cross‐sectional design limits the ability to establish causal relationships between the examined factors and the implementation of microaspiration prevention. Second, the data were based on nurses' self‐reports and may therefore be susceptible to social desirability bias if reported behaviour aligned more closely with social expectations than with actual practice. This could have resulted in overly optimistic findings. To reduce this risk, questionnaires were anonymous, and the importance of truthful responses was emphasised before the survey. Third, the original adherence questionnaire did not include a separate ‘not applicable’ response option, and item‐level information on whether each respondent had an opportunity to perform each practice was not collected. Consequently, the 22‐item total score may not have fully distinguished clinically appropriate non‐performance owing to item inapplicability or lack of opportunity from non‐adherence when a practice was applicable and feasible. Although the applicability‐focused sensitivity analysis excluding all eight items identified as condition‐specific in Supporting Information 1 generally supported the bed‐capacity pattern observed in the primary analysis, the association with microaspiration‐prevention training was attenuated and was no longer statistically significant. This analysis cannot establish that the scoring approach fully accounted for item‐level applicability because applicability was not assessed separately for individual participants. Future studies should collect item‐level applicability information and develop and validate an appropriate scoring procedure for ‘not applicable’ responses. Fourth, nurses' knowledge of microaspiration prevention was not directly measured in this study. Therefore, the potential pathway linking ICU bed capacity, continuing education, knowledge reinforcement, and adherence could not be directly tested. Fifth, participants were recruited using convenience sampling through professional networks and ICU head nurses, which may have introduced selection and self‐selection bias. Hospitals and nurses who were more willing to participate or more interested in microaspiration prevention may have been disproportionately represented. In addition, all participating hospitals were located in four cities in Henan Province, China. Therefore, the sample may not be representative of all ICU nurses in Henan Province or other regions, healthcare systems, and clinical settings, and the findings should be generalised with caution. Future studies should use broader geographic coverage and probability‐based or more representative sampling strategies. Sixth, ICU bed capacity was an ICU‐level exposure, but the analysis included only 31 ICUs, with six or seven ICUs in each bed‐capacity category. Consequently, the category‐specific estimates may have limited precision, as reflected by the width of some confidence intervals. Although a finite‐cluster reference‐distribution sensitivity analysis was performed, these estimates should still be interpreted cautiously. Finally, although the questionnaire was informed by the TDF and DIBQ, the TDF was not prospectively applied as a fully prespecified framework for item development, domain mapping and analysis. In this study, the implementation‐related items were interpreted primarily on the basis of their relative item‐level scores, and their subsequent interpretation in relation to TDF domains relied on item content and relevant previous studies. Accordingly, assigning individual items to specific TDF domains involved interpretive judgement, which may limit the validity of domain attribution and the extent to which individual items align with the original TDF construct definitions. The TDF‐related findings should therefore be considered as exploratory rather than confirmatory. Future prospective TDF‐based studies should incorporate theory‐informed item development and predefined domain mapping to rigorously validate the behavioural determinants of microaspiration prevention.

## 6 Conclusion

Overall, ICU nurses' self‐reported adherence varied across recommended microaspiration‐prevention practices and domains. After accounting for clustering within 31 ICUs and 17 hospitals and adjusting for clinically relevant covariates, ICU bed capacity remained associated with adherence: adjusted adherence scores were lower in the 10–14, 15–19, and ≥ 20‐bed groups than in the ICUs with < 5 beds, whereas the 5–9‐bed group did not differ significantly. Evidence‐based practice training and microaspiration‐prevention training were independently associated with higher adherence. Generalised estimating equation analyses, the finite‐cluster reference‐distribution sensitivity analysis, and models with alternative age/experience specifications generally supported the primary bed‐capacity pattern. In the applicability‐focused 14‐item analyses excluding all eight items identified as condition‐specific in Supporting Information 1, the bed‐capacity coefficients remained similar in direction and evidence‐based practice training remained positively associated with the outcome, whereas microaspiration‐prevention training remained positive but was not statistically significant. These findings represent statistical associations rather than causal relationships. The relatively low‐ and high‐scoring implementation‐related items highlight areas for further investigation, but these findings should be considered descriptive and exploratory.

## Author Contributions

**Yongju Pei:** writing – original draft, project administration, investigation, methodology, funding acquisition, formal analysis, conceptualization. **Shuyu Fan:** validation, data curation, formal analysis. **Xinping Shi:** methodology, formal analysis, supervision, validation.

## Funding

The authors have nothing to report.

## Ethics Statement

This study was reviewed and approved by the Ethics Committee of Henan Provincial People's Hospital as part of the 2024 Evidence‐Based Nursing Practice Project approval process (Approval/Reference No. HNSYXZKT202423; approval date: July 30, 2024).

## Conflicts of Interest

The authors declare no conflicts of interest.

## Patient Consent

Informed consent was obtained from all subjects involved in the study.

## Supporting information


**Supplementary File 1** Source of items in the adherence to microaspiration prevention.

**Supplementary File 2** Adherence to the microaspiration prevention (*N* = 476).

**Supplementary File 3** Sensitivity analyses of the multivariable association with microaspiration‐prevention adherence.
**Table S1:** Three‐level mixed‐effects model using the 14‐item sensitivity outcome.
**Table S2:** Core effects across primary and sensitivity models.
**Table S3:** Random‐effects structure and model diagnostics.
**Figure S1:** Normal Q–Q plot of conditional residuals from the primary 22‐item three‐level mixed‐effects model.
**Figure S2:** Conditional residuals versus fitted values from the primary 22‐item three‐level mixed‐effects model.
**Table S4:** Hospital‐level leave‐one‐cluster‐out analysis for the primary 22‐item three‐level mixed‐effects model.
**Table S5:** ICU‐level leave‐one‐cluster‐out analysis for the primary 22‐item three‐level mixed‐effects model.

## Data Availability

The data that support the findings of this study are available from the corresponding author upon reasonable request.
