# Supplementary material for: Intensive Care Nurses' Self‐Reported Adherence to Evidence‐Informed Microaspiration‐Prevention Practices and Potential Implementation Determinants: A Multicenter Cross‐Sectional Study
Source: Nurs Open. 2026 Oct 21;13(10):e70914. doi: 10.1002/nop2.70914 (PMC13687589; doi:10.1002/nop2.70914)
Supplement: Supplementary file 1 [file NOP2-13-e70914-s001.docx]

**Supplementary File 3 - Sensitivity Analyses of the Multivariable Association With Microaspiration-Prevention Adherence**

All sensitivity analyses used the same covariate set as the primary adjusted model unless otherwise stated. Age and ICU-working-experience categories were assigned representative values in ascending category order (age: 23, 28, 33, 38, 43, and 48 years; ICU working experience: 2, 4, 6, 8, 10, 12, 14, 16, 18, and 20 years) and entered as continuous covariates in the primary model. The primary outcome was the 22-item total score, interpreted as self-reported implementation frequency under respondents’ actual clinical conditions. The condition-specific sensitivity outcome excluded all eight items identified as condition-specific in Supplementary File 1 (Items 7, 11, 12, 13, 14, 15, 16, and 22) and used the mean of the remaining 14 items. This outcome was examined only as a robustness analysis and did not replace the primary 22-item outcome. Generalized estimating equation analyses used the ICU as the clustering unit, a Gaussian distribution with identity link, exchangeable and independent working correlation structures, and cluster-robust standard errors. A finite-cluster reference-distribution sensitivity analysis used t(30) reference distributions for individual coefficients and F(4,30) for the overall bed-capacity test.

Diagnostics were conducted for the primary 22-item three-level mixed-effects model. Conditional residuals, defined as observed values minus fitted values including the random effects, were assessed using a Q–Q plot, skewness, kurtosis, the Jarque–Bera test, and a residual-versus-fitted-value plot. Cluster-level influence was assessed by refitting the model after omitting one hospital or one ICU at a time. For each refitted model, convergence status and changes in the direction and statistical significance of five core coefficients were recorded: the 10–14-bed, 15–19-bed, and ≥20-bed contrasts relative to <5 beds and the evidence-based practice training and microaspiration-prevention training coefficients. Cook’s distance and the Breusch–Pagan test were calculated from an auxiliary ordinary least-squares model and were not treated as formal diagnostics of the three-level mixed-effects model. No observations were excluded solely on the basis of the auxiliary Cook’s distance threshold.

# **Table S1. Three-level mixed-effects model using the 14-item sensitivity outcome**

| **Variables** | **B** | **SE** | **95% CI** | **p** |
| --- | --- | --- | --- | --- |
| Constant | 4.397 | 0.325 | 3.760 to 5.035 | <0.001 |
| 5–9 beds vs. <5 beds | −0.035 | 0.135 | −0.300 to 0.230 | 0.798 |
| 10–14 beds vs. <5 beds | −0.320 | 0.136 | −0.587 to −0.054 | 0.018 |
| 15–19 beds vs. <5 beds | −0.334 | 0.167 | −0.662 to −0.006 | 0.046 |
| ≥20 beds vs. <5 beds | −0.314 | 0.157 | −0.622 to −0.005 | 0.046 |
| Medical/cardiopulmonary specialty ICU vs. general ICU | 0.122 | 0.159 | −0.191 to 0.434 | 0.446 |
| Surgical/neurosurgical ICU vs. general ICU | −0.139 | 0.152 | −0.437 to 0.159 | 0.361 |
| Emergency/other adult ICU vs. general ICU | −0.080 | 0.157 | −0.388 to 0.228 | 0.613 |
| Bachelor’s degree vs. junior college or below | −0.071 | 0.107 | −0.281 to 0.139 | 0.507 |
| Master’s degree vs. junior college or below | 0.285 | 0.109 | 0.072 to 0.499 | 0.009 |
| Doctoral degree vs. junior college or below | 0.445 | 0.151 | 0.149 to 0.741 | 0.003 |
| Tertiary vs. secondary hospital | 0.007 | 0.134 | −0.254 to 0.269 | 0.956 |
| Age representative value (per year) | −0.035 | 0.010 | −0.055 to −0.015 | <0.001 |
| ICU working experience representative value (per year) | 0.025 | 0.016 | −0.006 to 0.056 | 0.114 |
| Evidence-based practice training: yes vs. no | 0.202 | 0.085 | 0.035 to 0.369 | 0.018 |
| Microaspiration-prevention training: yes vs. no | 0.144 | 0.084 | −0.020 to 0.309 | 0.085 |

Bed capacity overall: Wald χ²(4)=10.276, p=0.036.

# **Table S2. Core effects across primary and sensitivity models**

| **Model** | **Bed overall p** | **5–9 beds vs. <5** | **10–14 beds vs. <5** | **15–19 beds vs. <5** | **≥20 beds vs. <5** | **EBP training** | **Microaspiration training** |
| --- | --- | --- | --- | --- | --- | --- | --- |
| Primary three-level mixed model | 0.010 | −0.772 (−6.249, 4.705); p=0.782 | −7.161 (−12.648, −1.674); p=0.011 | −8.383 (−15.275, −1.491); p=0.017 | −7.101 (−13.518, −0.684); p=0.030 | 3.971 (0.515, 7.426); p=0.024 | 3.947 (0.559, 7.335); p=0.022 |
| 14-item mixed model | 0.036 | −0.035 (−0.300, 0.230); p=0.798 | −0.320 (−0.587, −0.054); p=0.018 | −0.334 (−0.662, −0.006); p=0.046 | −0.314 (−0.622, −0.005); p=0.046 | 0.202 (0.035, 0.369); p=0.018 | 0.144 (−0.020, 0.309); p=0.085 |
| GEE exchangeable, 22-item | 0.015 | 0.644 (−6.009, 7.296); p=0.850 | −7.953 (−13.739, −2.168); p=0.007 | −7.923 (−14.740, −1.106); p=0.023 | −7.051 (−13.248, −0.854); p=0.026 | 3.490 (0.346, 6.633); p=0.030 | 4.041 (0.318, 7.764); p=0.033 |
| GEE independent, 22-item | 0.019 | 0.658 (−6.024, 7.340); p=0.847 | −7.933 (−13.910, −1.956); p=0.009 | −7.891 (−14.901, −0.882); p=0.027 | −6.935 (−13.305, −0.564); p=0.033 | 3.733 (0.549, 6.917); p=0.022 | 3.869 (0.077, 7.661); p=0.046 |
| GEE small-cluster inference, 22-item | 0.030 | 0.644 (−6.288, 7.575); p=0.851 | −7.953 (−13.981, −1.925); p=0.011 | −7.923 (−15.027, −0.820); p=0.030 | −7.051 (−13.509, −0.594); p=0.033 | 3.490 (0.214, 6.765); p=0.038 | 4.041 (0.161, 7.920); p=0.042 |
| GEE exchangeable, 14-item | 0.026 | 0.023 (−0.279, 0.325); p=0.883 | −0.368 (−0.635, −0.101); p=0.007 | −0.329 (−0.648, −0.011); p=0.043 | −0.320 (−0.614, −0.026); p=0.033 | 0.182 (0.020, 0.345); p=0.028 | 0.147 (−0.046, 0.340); p=0.136 |
| GEE independent, 14-item | 0.033 | 0.024 (−0.279, 0.326); p=0.878 | −0.367 (−0.644, −0.091); p=0.009 | −0.326 (−0.652, −0.001); p=0.050 | −0.315 (−0.617, −0.013); p=0.041 | 0.191 (0.027, 0.355); p=0.022 | 0.138 (−0.056, 0.333); p=0.163 |
| GEE finite-cluster reference distribution, 14-item | 0.045 | 0.023 (−0.292, 0.337); p=0.884 | −0.368 (−0.647, −0.089); p=0.011 | −0.329 (−0.661, 0.003); p=0.052 | −0.320 (−0.627, −0.013); p=0.041 | 0.182 (0.013, 0.351); p=0.036 | 0.147 (−0.054, 0.348); p=0.146 |
| Age/experience coarse categories | 0.008 | −0.991 (−6.493, 4.511); p=0.724 | −7.241 (−12.752, −1.731); p=0.010 | −8.844 (−15.752, −1.936); p=0.012 | −7.474 (−13.912, −1.037); p=0.023 | 3.810 (0.307, 7.313); p=0.033 | 4.042 (0.642, 7.443); p=0.020 |
| Experience orthogonalized to age | 0.010 | −0.772 (−6.249, 4.705); p=0.782 | −7.161 (−12.648, −1.674); p=0.011 | −8.383 (−15.275, −1.491); p=0.017 | −7.101 (−13.518, −0.684); p=0.030 | 3.971 (0.515, 7.426); p=0.024 | 3.947 (0.559, 7.335); p=0.022 |
| Age omitted | 0.009 | −0.701 (−6.177, 4.776); p=0.802 | −7.214 (−12.741, −1.686); p=0.011 | −8.374 (−15.289, −1.460); p=0.018 | −7.443 (−13.893, −0.994); p=0.024 | 3.649 (0.164, 7.133); p=0.040 | 4.227 (0.812, 7.642); p=0.015 |
| ICU experience omitted | 0.010 | −0.728 (−6.192, 4.735); p=0.794 | −7.239 (−12.722, −1.757); p=0.010 | −8.197 (−15.071, −1.322); p=0.019 | −7.065 (−13.479, −0.652); p=0.031 | 3.723 (0.298, 7.148); p=0.033 | 3.965 (0.576, 7.353); p=0.022 |

**Note:** Values for individual effects are adjusted unstandardized coefficients (B) with 95% confidence intervals, followed by p values. The 14-item outcome is reported on the original 1–5 mean-item scale. For the mixed-effects and standard GEE models, “Bed overall p” is from a four-degree-of-freedom Wald chi-square test for ICU bed capacity; for the finite-cluster reference-distribution sensitivity analyses, the overall bed-capacity test used F(4,30). Individual coefficients in the finite-cluster reference-distribution sensitivity analyses used t(30) reference distributions.

# **Table S3. Random-effects structure and model diagnostics**

| **Item** | **Result** | **Interpretation** |
| --- | --- | --- |
| Hospital random-intercept variance (REML primary model) | 24.762 | Hospital-level clustering |
| ICU incremental random-intercept variance (REML primary model) | 0.000004 | Near-zero incremental ICU-level variance |
| Residual variance | 260.303 | Level-1 residual variance |
| Hospital-level ICC | 8.69% | Estimated within-hospital correlation |
| Combined hospital-plus-ICU ICC | 8.69% | Estimated correlation for nurses in the same ICU |
| Three-level model convergence | Yes | Primary model converged |
| Mean / maximum VIF | 2.22 / 5.44 | Moderate collinearity; alternative age/experience analyses were consistent |
| ML three-level vs. hospital-only model | Log likelihood −2001.607 for both; AIC 4041.214 vs. 4039.214 | ICU incremental variance was near the boundary |
| Conditional residual skewness, primary 22-item model | −0.43 | Slight left skewness |
| Conditional residual kurtosis, primary 22-item model | 2.6 | Interpreted together with the Q–Q plot and Jarque–Bera test |
| Jarque–Bera test | χ²=17.76, p<0.001 | Departure from residual normality |
| Conditional residual-versus-fitted-value plot | Visible structure and unequal residual variance across fitted values | Departure from homoscedasticity |
| Hospital-level LOCO analysis | 15 of 17 refitted models met the convergence criterion; maximum \|ΔB\|=2.835 for the 10–14-bed contrast after deletion of H13; no directional reversal was observed in the available estimates for the five examined core coefficients | Statistical significance changed in some refits; nonconverged refits are identified in Table S4 |
| ICU-level LOCO analysis | 29 of 31 refitted models met the convergence criterion; maximum \|ΔB\|=2.689 for the 15–19-bed contrast after deletion of I21; no directional reversal was observed in the available estimates for the five examined core coefficients | Statistical significance changed in some refits; nonconverged refits are identified in Table S5 |
| Cook’s distance, observation-level OLS auxiliary assessment | 16 of 476 observations exceeded the 4/n screening threshold; maximum=0.019 | Auxiliary screening only; no observations were excluded |
| Breusch–Pagan test, OLS auxiliary assessment | χ²=35.46, p=0.002 | Auxiliary information only; not a formal diagnostic of the three-level mixed-effects model |

**Note:** The residual and influence diagnostics were conducted for the primary 22-item three-level mixed-effects model. The LOCO analyses examined five core coefficients: the 10–14-bed, 15–19-bed, and ≥20-bed contrasts relative to <5 beds and the evidence-based practice training and microaspiration-prevention training coefficients. ΔB was calculated as the coefficient after cluster deletion minus the corresponding full-sample coefficient. H13 and I21 are anonymized hospital and ICU codes, respectively. Complete hospital- and ICU-level LOCO results, including convergence status, are presented in Tables S4 and S5. Cook’s distance and the Breusch–Pagan test were calculated from an auxiliary OLS model and were not treated as formal diagnostics of the three-level mixed-effects model. The 4/n value was used only as an auxiliary screening threshold for Cook’s distance, and no observations were excluded solely on this basis. AIC, Akaike information criterion; ICC, intraclass correlation coefficient; ICU, intensive care unit; LOCO, leave-one-cluster-out; ML, maximum likelihood; OLS, ordinary least squares; REML, restricted maximum likelihood; VIF, variance inflation factor.

# **Figure S1. Normal Q–Q plot of conditional residuals from the primary 22-item three-level mixed-effects model.**

**Note:** Conditional residuals were calculated as observed values minus fitted values including the random effects. Points represent the ordered conditional residuals plotted against the theoretical quantiles of a standard normal distribution, and the solid line represents the reference line for normality. Skewness was −0.43, kurtosis was 2.60, and the Jarque–Bera test indicated departure from normality (χ²=17.76, p<0.001).

# **Figure S2. Conditional residuals versus fitted values from the primary 22-item three-level mixed-effects model.**

**Note:** The horizontal reference line indicates a residual value of zero. The plot showed visible structure and unequal residual variance across fitted values. The Breusch–Pagan test calculated from an auxiliary ordinary least-squares model yielded χ²=35.46 and p=0.002; this test was treated as auxiliary information and not as a formal diagnostic of the three-level mixed-effects model.

# **Table S4. Hospital-level leave-one-cluster-out analysis for the primary 22-item three-level mixed-effects model**

| **Omitted cluster** | **Sample size after omission** | **Converged** | **Coefficient** | **Full-sample B** | **Full-sample p** | **B after omission** | **SE after omission** | **p after omission** | **ΔB** | **Direction change** | **Significance change** |
| --- | --- | --- | --- | --- | --- | --- | --- | --- | --- | --- | --- |
| H01 | 449 | Yes | 10–14 beds versus <5 beds | -7.1610 | 0.0105 | -7.2973 | 3.5228 | 0.0383 | -0.1363 | No | No |
| H01 | 449 | Yes | 15–19 beds versus <5 beds | -8.3828 | 0.0171 | -8.1228 | 4.2071 | 0.0535 | 0.2599 | No | Yes |
| H01 | 449 | Yes | ≥20 beds versus <5 beds | -7.1006 | 0.0301 | -6.7058 | 4.4419 | 0.1311 | 0.3948 | No | Yes |
| H01 | 449 | Yes | Evidence-based practice training: yes versus no | 3.9708 | 0.0243 | 3.8266 | 1.8033 | 0.0338 | -0.1442 | No | No |
| H01 | 449 | Yes | Microaspiration-prevention training: yes versus no | 3.9468 | 0.0224 | 4.5174 | 1.7931 | 0.0118 | 0.5706 | No | No |
| H02 | 451 | Yes | 10–14 beds versus <5 beds | -7.1610 | 0.0105 | -7.5406 | 3.2789 | 0.0215 | -0.3796 | No | No |
| H02 | 451 | Yes | 15–19 beds versus <5 beds | -8.3828 | 0.0171 | -11.0543 | 4.5636 | 0.0154 | -2.6715 | No | No |
| H02 | 451 | Yes | ≥20 beds versus <5 beds | -7.1006 | 0.0301 | -8.0774 | 3.8179 | 0.0344 | -0.9769 | No | No |
| H02 | 451 | Yes | Evidence-based practice training: yes versus no | 3.9708 | 0.0243 | 2.7906 | 1.8064 | 0.1224 | -1.1802 | No | Yes |
| H02 | 451 | Yes | Microaspiration-prevention training: yes versus no | 3.9468 | 0.0224 | 3.7183 | 1.7603 | 0.0347 | -0.2284 | No | No |
| H03 | 445 | Yes | 10–14 beds versus <5 beds | -7.1610 | 0.0105 | -7.9578 | 3.1643 | 0.0119 | -0.7968 | No | No |
| H03 | 445 | Yes | 15–19 beds versus <5 beds | -8.3828 | 0.0171 | -9.5955 | 4.1235 | 0.0200 | -1.2127 | No | No |
| H03 | 445 | Yes | ≥20 beds versus <5 beds | -7.1006 | 0.0301 | -7.6159 | 3.6192 | 0.0354 | -0.5154 | No | No |
| H03 | 445 | Yes | Evidence-based practice training: yes versus no | 3.9708 | 0.0243 | 3.4570 | 1.8358 | 0.0597 | -0.5138 | No | Yes |
| H03 | 445 | Yes | Microaspiration-prevention training: yes versus no | 3.9468 | 0.0224 | 3.9779 | 1.8151 | 0.0284 | 0.0312 | No | No |
| H04 | 450 | Yes | 10–14 beds versus <5 beds | -7.1610 | 0.0105 | -7.4601 | 3.2417 | 0.0214 | -0.2991 | No | No |
| H04 | 450 | Yes | 15–19 beds versus <5 beds | -8.3828 | 0.0171 | -7.2951 | 4.1749 | 0.0806 | 1.0877 | No | Yes |
| H04 | 450 | Yes | ≥20 beds versus <5 beds | -7.1006 | 0.0301 | -7.0158 | 3.7134 | 0.0589 | 0.0848 | No | Yes |
| H04 | 450 | Yes | Evidence-based practice training: yes versus no | 3.9708 | 0.0243 | 2.8072 | 1.8301 | 0.1250 | -1.1636 | No | Yes |
| H04 | 450 | Yes | Microaspiration-prevention training: yes versus no | 3.9468 | 0.0224 | 4.5077 | 1.7885 | 0.0117 | 0.5609 | No | No |
| H05 | 447 | Yes | 10–14 beds versus <5 beds | -7.1610 | 0.0105 | -7.1921 | 3.3093 | 0.0298 | -0.0311 | No | No |
| H05 | 447 | Yes | 15–19 beds versus <5 beds | -8.3828 | 0.0171 | -8.0450 | 4.1767 | 0.0541 | 0.3378 | No | Yes |
| H05 | 447 | Yes | ≥20 beds versus <5 beds | -7.1006 | 0.0301 | -7.1381 | 3.9797 | 0.0729 | -0.0376 | No | Yes |
| H05 | 447 | Yes | Evidence-based practice training: yes versus no | 3.9708 | 0.0243 | 3.3852 | 1.8002 | 0.0600 | -0.5856 | No | Yes |
| H05 | 447 | Yes | Microaspiration-prevention training: yes versus no | 3.9468 | 0.0224 | 3.5632 | 1.7725 | 0.0444 | -0.3836 | No | No |
| H06 | 441 | Yes | 10–14 beds versus <5 beds | -7.1610 | 0.0105 | -6.6800 | 3.4366 | 0.0519 | 0.4810 | No | Yes |
| H06 | 441 | Yes | 15–19 beds versus <5 beds | -8.3828 | 0.0171 | -7.6608 | 4.0190 | 0.0566 | 0.7220 | No | Yes |
| H06 | 441 | Yes | ≥20 beds versus <5 beds | -7.1006 | 0.0301 | -6.8240 | 3.7548 | 0.0692 | 0.2765 | No | Yes |
| H06 | 441 | Yes | Evidence-based practice training: yes versus no | 3.9708 | 0.0243 | 3.9320 | 1.8589 | 0.0344 | -0.0388 | No | No |
| H06 | 441 | Yes | Microaspiration-prevention training: yes versus no | 3.9468 | 0.0224 | 4.3641 | 1.8116 | 0.0160 | 0.4173 | No | No |
| H07 | 440 | Yes | 10–14 beds versus <5 beds | -7.1610 | 0.0105 | -7.1007 | 3.5951 | 0.0483 | 0.0603 | No | No |
| H07 | 440 | Yes | 15–19 beds versus <5 beds | -8.3828 | 0.0171 | -9.8535 | 3.9619 | 0.0129 | -1.4707 | No | No |
| H07 | 440 | Yes | ≥20 beds versus <5 beds | -7.1006 | 0.0301 | -8.4592 | 3.8804 | 0.0293 | -1.3586 | No | No |
| H07 | 440 | Yes | Evidence-based practice training: yes versus no | 3.9708 | 0.0243 | 3.5411 | 1.7791 | 0.0466 | -0.4298 | No | No |
| H07 | 440 | Yes | Microaspiration-prevention training: yes versus no | 3.9468 | 0.0224 | 3.9127 | 1.7465 | 0.0251 | -0.0341 | No | No |
| H08 | 446 | Yes | 10–14 beds versus <5 beds | -7.1610 | 0.0105 | -9.0275 | 2.9853 | 0.0025 | -1.8665 | No | No |
| H08 | 446 | Yes | 15–19 beds versus <5 beds | -8.3828 | 0.0171 | -6.3323 | 3.5991 | 0.0785 | 2.0505 | No | Yes |
| H08 | 446 | Yes | ≥20 beds versus <5 beds | -7.1006 | 0.0301 | -5.3039 | 3.5046 | 0.1302 | 1.7966 | No | Yes |
| H08 | 446 | Yes | Evidence-based practice training: yes versus no | 3.9708 | 0.0243 | 4.6948 | 1.8621 | 0.0117 | 0.7240 | No | No |
| H08 | 446 | Yes | Microaspiration-prevention training: yes versus no | 3.9468 | 0.0224 | 3.9193 | 1.8050 | 0.0299 | -0.0275 | No | No |
| H09 | 460 | Yes | 10–14 beds versus <5 beds | -7.1610 | 0.0105 | -7.4022 | 2.7938 | 0.0081 | -0.2412 | No | No |
| H09 | 460 | Yes | 15–19 beds versus <5 beds | -8.3828 | 0.0171 | -7.3215 | 3.5939 | 0.0416 | 1.0613 | No | No |
| H09 | 460 | Yes | ≥20 beds versus <5 beds | -7.1006 | 0.0301 | -7.2182 | 3.2645 | 0.0270 | -0.1176 | No | No |
| H09 | 460 | Yes | Evidence-based practice training: yes versus no | 3.9708 | 0.0243 | 4.0945 | 1.7906 | 0.0222 | 0.1236 | No | No |
| H09 | 460 | Yes | Microaspiration-prevention training: yes versus no | 3.9468 | 0.0224 | 4.3077 | 1.7565 | 0.0142 | 0.3609 | No | No |
| H10 | 461 | No | 10–14 beds versus <5 beds | -7.1610 | 0.0105 | -7.1735 | 2.8145 | 0.0108 | -0.0125 | No | No |
| H10 | 461 | No | 15–19 beds versus <5 beds | -8.3828 | 0.0171 | -9.3959 | 3.6027 | 0.0091 | -1.0131 | No | No |
| H10 | 461 | No | ≥20 beds versus <5 beds | -7.1006 | 0.0301 | -7.1151 | 3.2988 | 0.0310 | -0.0145 | No | No |
| H10 | 461 | No | Evidence-based practice training: yes versus no | 3.9708 | 0.0243 | 3.4219 | 1.7988 | 0.0571 | -0.5489 | No | Yes |
| H10 | 461 | No | Microaspiration-prevention training: yes versus no | 3.9468 | 0.0224 | 3.6109 | 1.7705 | 0.0414 | -0.3359 | No | No |
| H11 | 446 | Yes | 10–14 beds versus <5 beds | -7.1610 | 0.0105 | -7.9809 | 3.0609 | 0.0091 | -0.8199 | No | No |
| H11 | 446 | Yes | 15–19 beds versus <5 beds | -8.3828 | 0.0171 | -7.2803 | 3.6263 | 0.0447 | 1.1025 | No | No |
| H11 | 446 | Yes | ≥20 beds versus <5 beds | -7.1006 | 0.0301 | -9.3737 | 3.7012 | 0.0113 | -2.2732 | No | No |
| H11 | 446 | Yes | Evidence-based practice training: yes versus no | 3.9708 | 0.0243 | 3.9850 | 1.8080 | 0.0275 | 0.0142 | No | No |
| H11 | 446 | Yes | Microaspiration-prevention training: yes versus no | 3.9468 | 0.0224 | 4.0818 | 1.7865 | 0.0223 | 0.1350 | No | No |
| H12 | 464 | Yes | 10–14 beds versus <5 beds | -7.1610 | 0.0105 | -7.0809 | 3.2341 | 0.0286 | 0.0801 | No | No |
| H12 | 464 | Yes | 15–19 beds versus <5 beds | -8.3828 | 0.0171 | -8.1393 | 3.8991 | 0.0368 | 0.2435 | No | No |
| H12 | 464 | Yes | ≥20 beds versus <5 beds | -7.1006 | 0.0301 | -7.7553 | 3.9912 | 0.0520 | -0.6547 | No | Yes |
| H12 | 464 | Yes | Evidence-based practice training: yes versus no | 3.9708 | 0.0243 | 3.6991 | 1.7996 | 0.0398 | -0.2717 | No | No |
| H12 | 464 | Yes | Microaspiration-prevention training: yes versus no | 3.9468 | 0.0224 | 3.5081 | 1.7676 | 0.0472 | -0.4386 | No | No |
| H13 | 441 | Yes | 10–14 beds versus <5 beds | -7.1610 | 0.0105 | -4.3262 | 3.2012 | 0.1766 | 2.8348 | No | Yes |
| H13 | 441 | Yes | 15–19 beds versus <5 beds | -8.3828 | 0.0171 | -6.1968 | 3.7463 | 0.0981 | 2.1859 | No | Yes |
| H13 | 441 | Yes | ≥20 beds versus <5 beds | -7.1006 | 0.0301 | -4.5089 | 3.5871 | 0.2088 | 2.5917 | No | Yes |
| H13 | 441 | Yes | Evidence-based practice training: yes versus no | 3.9708 | 0.0243 | 4.4424 | 1.8126 | 0.0142 | 0.4716 | No | No |
| H13 | 441 | Yes | Microaspiration-prevention training: yes versus no | 3.9468 | 0.0224 | 3.5764 | 1.8083 | 0.0479 | -0.3703 | No | No |
| H14 | 448 | Yes | 10–14 beds versus <5 beds | -7.1610 | 0.0105 | -7.8933 | 3.1621 | 0.0126 | -0.7324 | No | No |
| H14 | 448 | Yes | 15–19 beds versus <5 beds | -8.3828 | 0.0171 | -8.8256 | 3.7519 | 0.0187 | -0.4428 | No | No |
| H14 | 448 | Yes | ≥20 beds versus <5 beds | -7.1006 | 0.0301 | -5.8611 | 3.6121 | 0.1047 | 1.2395 | No | Yes |
| H14 | 448 | Yes | Evidence-based practice training: yes versus no | 3.9708 | 0.0243 | 3.6634 | 1.8236 | 0.0445 | -0.3074 | No | No |
| H14 | 448 | Yes | Microaspiration-prevention training: yes versus no | 3.9468 | 0.0224 | 3.8895 | 1.7658 | 0.0276 | -0.0573 | No | No |
| H15 | 442 | No | 10–14 beds versus <5 beds | -7.1610 | 0.0105 | -7.3250 | 2.9107 | 0.0119 | -0.1640 | No | No |
| H15 | 442 | No | 15–19 beds versus <5 beds | -8.3828 | 0.0171 | -8.4971 | 3.5997 | 0.0182 | -0.1143 | No | No |
| H15 | 442 | No | ≥20 beds versus <5 beds | -7.1006 | 0.0301 | -7.2215 | 3.3399 | 0.0306 | -0.1210 | No | No |
| H15 | 442 | No | Evidence-based practice training: yes versus no | 3.9708 | 0.0243 | 3.8343 | 1.8271 | 0.0359 | -0.1365 | No | No |
| H15 | 442 | No | Microaspiration-prevention training: yes versus no | 3.9468 | 0.0224 | 4.2054 | 1.7976 | 0.0193 | 0.2587 | No | No |
| H16 | 442 | Yes | 10–14 beds versus <5 beds | -7.1610 | 0.0105 | -6.9050 | 3.6658 | 0.0596 | 0.2560 | No | Yes |
| H16 | 442 | Yes | 15–19 beds versus <5 beds | -8.3828 | 0.0171 | -8.6125 | 3.9399 | 0.0288 | -0.2297 | No | No |
| H16 | 442 | Yes | ≥20 beds versus <5 beds | -7.1006 | 0.0301 | -7.2776 | 3.8014 | 0.0556 | -0.1770 | No | Yes |
| H16 | 442 | Yes | Evidence-based practice training: yes versus no | 3.9708 | 0.0243 | 4.4964 | 1.8009 | 0.0125 | 0.5256 | No | No |
| H16 | 442 | Yes | Microaspiration-prevention training: yes versus no | 3.9468 | 0.0224 | 3.9288 | 1.7453 | 0.0244 | -0.0180 | No | No |
| H17 | 443 | Yes | 10–14 beds versus <5 beds | -7.1610 | 0.0105 | -5.6768 | 3.6575 | 0.1206 | 1.4842 | No | Yes |
| H17 | 443 | Yes | 15–19 beds versus <5 beds | -8.3828 | 0.0171 | -7.3395 | 3.9853 | 0.0655 | 1.0432 | No | Yes |
| H17 | 443 | Yes | ≥20 beds versus <5 beds | -7.1006 | 0.0301 | -6.0584 | 3.7642 | 0.1075 | 1.0422 | No | Yes |
| H17 | 443 | Yes | Evidence-based practice training: yes versus no | 3.9708 | 0.0243 | 4.2079 | 1.8408 | 0.0223 | 0.2371 | No | No |
| H17 | 443 | Yes | Microaspiration-prevention training: yes versus no | 3.9468 | 0.0224 | 4.2280 | 1.7914 | 0.0183 | 0.2813 | No | No |

**Note:** Each model was refitted after omission of one hospital. The analysis examined five core coefficients: the 10–14-bed, 15–19-bed, and ≥20-bed contrasts relative to <5 beds and the evidence-based practice training and microaspiration-prevention training coefficients. ΔB was calculated as the coefficient after cluster omission minus the corresponding full-sample coefficient. Direction change indicates a reversal in the sign of the coefficient, and significance change indicates a change in statistical significance at α=0.05. Models omitting H10 and H15 did not meet the convergence criterion. Estimates from models that did not meet the convergence criterion are presented for completeness but should not be interpreted as reliable inferential results. B, unstandardized coefficient; ICU, intensive care unit; LOCO, leave-one-cluster-out; SE, standard error.

# **Table S5. ICU-level leave-one-cluster-out analysis for the primary 22-item three-level mixed-effects model**

| **Omitted cluster** | **Sample size after omission** | **Converged** | **Coefficient** | **Full-sample B** | **Full-sample p** | **B after omission** | **SE after omission** | **p after omission** | **ΔB** | **Direction change** | **Significance change** |
| --- | --- | --- | --- | --- | --- | --- | --- | --- | --- | --- | --- |
| I01 | 464 | Yes | 10–14 beds versus <5 beds | -7.1610 | 0.0105 | -7.6039 | 3.2478 | 0.0192 | -0.4429 | No | No |
| I01 | 464 | Yes | 15–19 beds versus <5 beds | -8.3828 | 0.0171 | -9.1197 | 4.2671 | 0.0326 | -0.7369 | No | No |
| I01 | 464 | Yes | ≥20 beds versus <5 beds | -7.1006 | 0.0301 | -7.6022 | 3.8032 | 0.0456 | -0.5016 | No | No |
| I01 | 464 | Yes | Evidence-based practice training: yes versus no | 3.9708 | 0.0243 | 3.3487 | 1.7903 | 0.0614 | -0.6221 | No | Yes |
| I01 | 464 | Yes | Microaspiration-prevention training: yes versus no | 3.9468 | 0.0224 | 3.8569 | 1.7358 | 0.0263 | -0.0899 | No | No |
| I02 | 464 | Yes | 10–14 beds versus <5 beds | -7.1610 | 0.0105 | -7.3993 | 3.4228 | 0.0306 | -0.2383 | No | No |
| I02 | 464 | Yes | 15–19 beds versus <5 beds | -8.3828 | 0.0171 | -8.2979 | 4.0843 | 0.0422 | 0.0849 | No | No |
| I02 | 464 | Yes | ≥20 beds versus <5 beds | -7.1006 | 0.0301 | -7.2840 | 4.1552 | 0.0796 | -0.1834 | No | Yes |
| I02 | 464 | Yes | Evidence-based practice training: yes versus no | 3.9708 | 0.0243 | 3.9628 | 1.7762 | 0.0257 | -0.0080 | No | No |
| I02 | 464 | Yes | Microaspiration-prevention training: yes versus no | 3.9468 | 0.0224 | 4.3777 | 1.7600 | 0.0129 | 0.4309 | No | No |
| I03 | 464 | Yes | 10–14 beds versus <5 beds | -7.1610 | 0.0105 | -7.7716 | 3.2164 | 0.0157 | -0.6106 | No | No |
| I03 | 464 | Yes | 15–19 beds versus <5 beds | -8.3828 | 0.0171 | -9.4334 | 4.1697 | 0.0237 | -1.0507 | No | No |
| I03 | 464 | Yes | ≥20 beds versus <5 beds | -7.1006 | 0.0301 | -7.7269 | 3.6682 | 0.0352 | -0.6263 | No | No |
| I03 | 464 | Yes | Evidence-based practice training: yes versus no | 3.9708 | 0.0243 | 3.6432 | 1.8055 | 0.0436 | -0.3276 | No | No |
| I03 | 464 | Yes | Microaspiration-prevention training: yes versus no | 3.9468 | 0.0224 | 3.8469 | 1.7673 | 0.0295 | -0.0999 | No | No |
| I04 | 459 | Yes | 10–14 beds versus <5 beds | -7.1610 | 0.0105 | -8.2839 | 3.6640 | 0.0238 | -1.1229 | No | No |
| I04 | 459 | Yes | 15–19 beds versus <5 beds | -8.3828 | 0.0171 | -8.8507 | 4.0215 | 0.0277 | -0.4680 | No | No |
| I04 | 459 | Yes | ≥20 beds versus <5 beds | -7.1006 | 0.0301 | -7.6600 | 3.8831 | 0.0485 | -0.5594 | No | No |
| I04 | 459 | Yes | Evidence-based practice training: yes versus no | 3.9708 | 0.0243 | 3.8776 | 1.7873 | 0.0300 | -0.0932 | No | No |
| I04 | 459 | Yes | Microaspiration-prevention training: yes versus no | 3.9468 | 0.0224 | 3.6743 | 1.7424 | 0.0350 | -0.2725 | No | No |
| I05 | 460 | Yes | 10–14 beds versus <5 beds | -7.1610 | 0.0105 | -5.8362 | 3.4897 | 0.0944 | 1.3248 | No | Yes |
| I05 | 460 | Yes | 15–19 beds versus <5 beds | -8.3828 | 0.0171 | -7.4944 | 3.9356 | 0.0569 | 0.8884 | No | Yes |
| I05 | 460 | Yes | ≥20 beds versus <5 beds | -7.1006 | 0.0301 | -6.1904 | 3.7254 | 0.0966 | 0.9102 | No | Yes |
| I05 | 460 | Yes | Evidence-based practice training: yes versus no | 3.9708 | 0.0243 | 4.1313 | 1.8037 | 0.0220 | 0.1605 | No | No |
| I05 | 460 | Yes | Microaspiration-prevention training: yes versus no | 3.9468 | 0.0224 | 4.0209 | 1.7785 | 0.0238 | 0.0741 | No | No |
| I06 | 460 | Yes | 10–14 beds versus <5 beds | -7.1610 | 0.0105 | -4.9107 | 3.1654 | 0.1208 | 2.2502 | No | Yes |
| I06 | 460 | Yes | 15–19 beds versus <5 beds | -8.3828 | 0.0171 | -6.3973 | 3.7106 | 0.0847 | 1.9855 | No | Yes |
| I06 | 460 | Yes | ≥20 beds versus <5 beds | -7.1006 | 0.0301 | -4.8377 | 3.5504 | 0.1730 | 2.2628 | No | Yes |
| I06 | 460 | Yes | Evidence-based practice training: yes versus no | 3.9708 | 0.0243 | 4.2664 | 1.7797 | 0.0165 | 0.2956 | No | No |
| I06 | 460 | Yes | Microaspiration-prevention training: yes versus no | 3.9468 | 0.0224 | 4.5328 | 1.7606 | 0.0100 | 0.5860 | No | No |
| I07 | 456 | Yes | 10–14 beds versus <5 beds | -7.1610 | 0.0105 | -8.1806 | 2.8349 | 0.0039 | -1.0196 | No | No |
| I07 | 456 | Yes | 15–19 beds versus <5 beds | -8.3828 | 0.0171 | -10.0320 | 3.5841 | 0.0051 | -1.6492 | No | No |
| I07 | 456 | Yes | ≥20 beds versus <5 beds | -7.1006 | 0.0301 | -8.4882 | 3.3419 | 0.0111 | -1.3876 | No | No |
| I07 | 456 | Yes | Evidence-based practice training: yes versus no | 3.9708 | 0.0243 | 3.6047 | 1.7763 | 0.0424 | -0.3661 | No | No |
| I07 | 456 | Yes | Microaspiration-prevention training: yes versus no | 3.9468 | 0.0224 | 4.2468 | 1.7554 | 0.0155 | 0.3000 | No | No |
| I08 | 456 | Yes | 10–14 beds versus <5 beds | -7.1610 | 0.0105 | -6.6973 | 3.2272 | 0.0380 | 0.4637 | No | No |
| I08 | 456 | Yes | 15–19 beds versus <5 beds | -8.3828 | 0.0171 | -7.7956 | 3.8629 | 0.0436 | 0.5872 | No | No |
| I08 | 456 | Yes | ≥20 beds versus <5 beds | -7.1006 | 0.0301 | -6.8329 | 3.6430 | 0.0607 | 0.2677 | No | Yes |
| I08 | 456 | Yes | Evidence-based practice training: yes versus no | 3.9708 | 0.0243 | 3.9161 | 1.8286 | 0.0322 | -0.0547 | No | No |
| I08 | 456 | Yes | Microaspiration-prevention training: yes versus no | 3.9468 | 0.0224 | 4.0413 | 1.8068 | 0.0253 | 0.0945 | No | No |
| I09 | 457 | Yes | 10–14 beds versus <5 beds | -7.1610 | 0.0105 | -7.9172 | 2.8667 | 0.0057 | -0.7562 | No | No |
| I09 | 457 | Yes | 15–19 beds versus <5 beds | -8.3828 | 0.0171 | -9.4897 | 3.7205 | 0.0108 | -1.1070 | No | No |
| I09 | 457 | Yes | ≥20 beds versus <5 beds | -7.1006 | 0.0301 | -7.4838 | 3.3289 | 0.0246 | -0.3832 | No | No |
| I09 | 457 | Yes | Evidence-based practice training: yes versus no | 3.9708 | 0.0243 | 3.7235 | 1.7914 | 0.0377 | -0.2473 | No | No |
| I09 | 457 | Yes | Microaspiration-prevention training: yes versus no | 3.9468 | 0.0224 | 4.0570 | 1.7734 | 0.0222 | 0.1102 | No | No |
| I10 | 457 | Yes | 10–14 beds versus <5 beds | -7.1610 | 0.0105 | -5.5168 | 3.0275 | 0.0684 | 1.6442 | No | Yes |
| I10 | 457 | Yes | 15–19 beds versus <5 beds | -8.3828 | 0.0171 | -7.3191 | 3.6134 | 0.0428 | 1.0637 | No | No |
| I10 | 457 | Yes | ≥20 beds versus <5 beds | -7.1006 | 0.0301 | -5.7255 | 3.4031 | 0.0925 | 1.3751 | No | Yes |
| I10 | 457 | Yes | Evidence-based practice training: yes versus no | 3.9708 | 0.0243 | 4.0270 | 1.7912 | 0.0246 | 0.0562 | No | No |
| I10 | 457 | Yes | Microaspiration-prevention training: yes versus no | 3.9468 | 0.0224 | 2.9602 | 1.7741 | 0.0952 | -0.9866 | No | Yes |
| I11 | 457 | Yes | 10–14 beds versus <5 beds | -7.1610 | 0.0105 | -7.7311 | 2.9485 | 0.0087 | -0.5701 | No | No |
| I11 | 457 | Yes | 15–19 beds versus <5 beds | -8.3828 | 0.0171 | -7.6604 | 3.5858 | 0.0327 | 0.7224 | No | No |
| I11 | 457 | Yes | ≥20 beds versus <5 beds | -7.1006 | 0.0301 | -8.4433 | 3.6201 | 0.0197 | -1.3427 | No | No |
| I11 | 457 | Yes | Evidence-based practice training: yes versus no | 3.9708 | 0.0243 | 3.9413 | 1.7826 | 0.0270 | -0.0295 | No | No |
| I11 | 457 | Yes | Microaspiration-prevention training: yes versus no | 3.9468 | 0.0224 | 4.1479 | 1.7522 | 0.0179 | 0.2011 | No | No |
| I12 | 457 | No | 10–14 beds versus <5 beds | -7.1610 | 0.0105 | -7.2680 | 2.8006 | 0.0095 | -0.1070 | No | No |
| I12 | 457 | No | 15–19 beds versus <5 beds | -8.3828 | 0.0171 | -7.3808 | 3.4754 | 0.0337 | 1.0020 | No | No |
| I12 | 457 | No | ≥20 beds versus <5 beds | -7.1006 | 0.0301 | -7.5150 | 3.2485 | 0.0207 | -0.4144 | No | No |
| I12 | 457 | No | Evidence-based practice training: yes versus no | 3.9708 | 0.0243 | 3.7421 | 1.8079 | 0.0385 | -0.2287 | No | No |
| I12 | 457 | No | Microaspiration-prevention training: yes versus no | 3.9468 | 0.0224 | 3.9634 | 1.7779 | 0.0258 | 0.0167 | No | No |
| I13 | 460 | Yes | 10–14 beds versus <5 beds | -7.1610 | 0.0105 | -6.4767 | 3.4989 | 0.0642 | 0.6843 | No | Yes |
| I13 | 460 | Yes | 15–19 beds versus <5 beds | -8.3828 | 0.0171 | -8.0500 | 3.9646 | 0.0423 | 0.3328 | No | No |
| I13 | 460 | Yes | ≥20 beds versus <5 beds | -7.1006 | 0.0301 | -6.7410 | 3.7787 | 0.0744 | 0.3596 | No | Yes |
| I13 | 460 | Yes | Evidence-based practice training: yes versus no | 3.9708 | 0.0243 | 3.9449 | 1.7654 | 0.0254 | -0.0259 | No | No |
| I13 | 460 | Yes | Microaspiration-prevention training: yes versus no | 3.9468 | 0.0224 | 3.6490 | 1.7220 | 0.0341 | -0.2978 | No | No |
| I14 | 461 | Yes | 10–14 beds versus <5 beds | -7.1610 | 0.0105 | -6.7703 | 3.4344 | 0.0487 | 0.3907 | No | No |
| I14 | 461 | Yes | 15–19 beds versus <5 beds | -8.3828 | 0.0171 | -8.2272 | 3.9468 | 0.0371 | 0.1556 | No | No |
| I14 | 461 | Yes | ≥20 beds versus <5 beds | -7.1006 | 0.0301 | -6.9803 | 3.7326 | 0.0615 | 0.1203 | No | Yes |
| I14 | 461 | Yes | Evidence-based practice training: yes versus no | 3.9708 | 0.0243 | 3.7412 | 1.7836 | 0.0359 | -0.2296 | No | No |
| I14 | 461 | Yes | Microaspiration-prevention training: yes versus no | 3.9468 | 0.0224 | 4.3545 | 1.7288 | 0.0118 | 0.4077 | No | No |
| I15 | 461 | Yes | 10–14 beds versus <5 beds | -7.1610 | 0.0105 | -8.9319 | 2.9894 | 0.0028 | -1.7709 | No | No |
| I15 | 461 | Yes | 15–19 beds versus <5 beds | -8.3828 | 0.0171 | -6.6780 | 3.5832 | 0.0624 | 1.7048 | No | Yes |
| I15 | 461 | Yes | ≥20 beds versus <5 beds | -7.1006 | 0.0301 | -4.8431 | 3.4764 | 0.1636 | 2.2575 | No | Yes |
| I15 | 461 | Yes | Evidence-based practice training: yes versus no | 3.9708 | 0.0243 | 4.2430 | 1.8051 | 0.0187 | 0.2722 | No | No |
| I15 | 461 | Yes | Microaspiration-prevention training: yes versus no | 3.9468 | 0.0224 | 4.4544 | 1.7650 | 0.0116 | 0.5076 | No | No |
| I16 | 459 | Yes | 10–14 beds versus <5 beds | -7.1610 | 0.0105 | -5.7416 | 3.6420 | 0.1149 | 1.4194 | No | Yes |
| I16 | 459 | Yes | 15–19 beds versus <5 beds | -8.3828 | 0.0171 | -7.5294 | 3.9513 | 0.0567 | 0.8534 | No | Yes |
| I16 | 459 | Yes | ≥20 beds versus <5 beds | -7.1006 | 0.0301 | -6.2342 | 3.8006 | 0.1009 | 0.8664 | No | Yes |
| I16 | 459 | Yes | Evidence-based practice training: yes versus no | 3.9708 | 0.0243 | 4.2819 | 1.7733 | 0.0158 | 0.3111 | No | No |
| I16 | 459 | Yes | Microaspiration-prevention training: yes versus no | 3.9468 | 0.0224 | 4.2641 | 1.7312 | 0.0138 | 0.3173 | No | No |
| I17 | 459 | Yes | 10–14 beds versus <5 beds | -7.1610 | 0.0105 | -6.4015 | 3.5321 | 0.0699 | 0.7595 | No | Yes |
| I17 | 459 | Yes | 15–19 beds versus <5 beds | -8.3828 | 0.0171 | -7.8478 | 3.8639 | 0.0423 | 0.5350 | No | No |
| I17 | 459 | Yes | ≥20 beds versus <5 beds | -7.1006 | 0.0301 | -6.6741 | 3.6462 | 0.0672 | 0.4264 | No | Yes |
| I17 | 459 | Yes | Evidence-based practice training: yes versus no | 3.9708 | 0.0243 | 3.8310 | 1.7938 | 0.0327 | -0.1398 | No | No |
| I17 | 459 | Yes | Microaspiration-prevention training: yes versus no | 3.9468 | 0.0224 | 4.2238 | 1.7367 | 0.0150 | 0.2770 | No | No |
| I18 | 460 | Yes | 10–14 beds versus <5 beds | -7.1610 | 0.0105 | -7.8920 | 3.1737 | 0.0129 | -0.7310 | No | No |
| I18 | 460 | Yes | 15–19 beds versus <5 beds | -8.3828 | 0.0171 | -8.1096 | 3.7866 | 0.0322 | 0.2732 | No | No |
| I18 | 460 | Yes | ≥20 beds versus <5 beds | -7.1006 | 0.0301 | -6.8918 | 3.5467 | 0.0520 | 0.2088 | No | Yes |
| I18 | 460 | Yes | Evidence-based practice training: yes versus no | 3.9708 | 0.0243 | 3.9636 | 1.7784 | 0.0258 | -0.0072 | No | No |
| I18 | 460 | Yes | Microaspiration-prevention training: yes versus no | 3.9468 | 0.0224 | 3.9258 | 1.7310 | 0.0233 | -0.0210 | No | No |
| I19 | 462 | Yes | 10–14 beds versus <5 beds | -7.1610 | 0.0105 | -7.2439 | 3.2378 | 0.0253 | -0.0829 | No | No |
| I19 | 462 | Yes | 15–19 beds versus <5 beds | -8.3828 | 0.0171 | -8.0326 | 4.0827 | 0.0491 | 0.3502 | No | No |
| I19 | 462 | Yes | ≥20 beds versus <5 beds | -7.1006 | 0.0301 | -7.2160 | 3.7108 | 0.0518 | -0.1154 | No | Yes |
| I19 | 462 | Yes | Evidence-based practice training: yes versus no | 3.9708 | 0.0243 | 3.6442 | 1.7843 | 0.0411 | -0.3266 | No | No |
| I19 | 462 | Yes | Microaspiration-prevention training: yes versus no | 3.9468 | 0.0224 | 3.8758 | 1.7510 | 0.0269 | -0.0710 | No | No |
| I20 | 463 | Yes | 10–14 beds versus <5 beds | -7.1610 | 0.0105 | -7.3836 | 3.1669 | 0.0197 | -0.2226 | No | No |
| I20 | 463 | Yes | 15–19 beds versus <5 beds | -8.3828 | 0.0171 | -7.5637 | 3.8732 | 0.0508 | 0.8191 | No | Yes |
| I20 | 463 | Yes | ≥20 beds versus <5 beds | -7.1006 | 0.0301 | -6.9199 | 3.6230 | 0.0561 | 0.1806 | No | Yes |
| I20 | 463 | Yes | Evidence-based practice training: yes versus no | 3.9708 | 0.0243 | 3.4096 | 1.7979 | 0.0579 | -0.5612 | No | Yes |
| I20 | 463 | Yes | Microaspiration-prevention training: yes versus no | 3.9468 | 0.0224 | 4.8640 | 1.7568 | 0.0056 | 0.9172 | No | No |
| I21 | 463 | Yes | 10–14 beds versus <5 beds | -7.1610 | 0.0105 | -7.5334 | 3.1513 | 0.0168 | -0.3724 | No | No |
| I21 | 463 | Yes | 15–19 beds versus <5 beds | -8.3828 | 0.0171 | -11.0721 | 4.4351 | 0.0125 | -2.6894 | No | No |
| I21 | 463 | Yes | ≥20 beds versus <5 beds | -7.1006 | 0.0301 | -8.0685 | 3.6739 | 0.0281 | -0.9679 | No | No |
| I21 | 463 | Yes | Evidence-based practice training: yes versus no | 3.9708 | 0.0243 | 3.2167 | 1.7750 | 0.0700 | -0.7541 | No | Yes |
| I21 | 463 | Yes | Microaspiration-prevention training: yes versus no | 3.9468 | 0.0224 | 3.9011 | 1.7504 | 0.0258 | -0.0457 | No | No |
| I22 | 463 | Yes | 10–14 beds versus <5 beds | -7.1610 | 0.0105 | -7.1372 | 3.2216 | 0.0267 | 0.0238 | No | No |
| I22 | 463 | Yes | 15–19 beds versus <5 beds | -8.3828 | 0.0171 | -8.6604 | 3.9577 | 0.0287 | -0.2776 | No | No |
| I22 | 463 | Yes | ≥20 beds versus <5 beds | -7.1006 | 0.0301 | -7.2608 | 3.6911 | 0.0492 | -0.1602 | No | No |
| I22 | 463 | Yes | Evidence-based practice training: yes versus no | 3.9708 | 0.0243 | 3.2553 | 1.8019 | 0.0708 | -0.7155 | No | Yes |
| I22 | 463 | Yes | Microaspiration-prevention training: yes versus no | 3.9468 | 0.0224 | 3.6465 | 1.7565 | 0.0379 | -0.3002 | No | No |
| I23 | 460 | Yes | 10–14 beds versus <5 beds | -7.1610 | 0.0105 | -7.4022 | 2.7938 | 0.0081 | -0.2412 | No | No |
| I23 | 460 | Yes | 15–19 beds versus <5 beds | -8.3828 | 0.0171 | -7.3215 | 3.5939 | 0.0416 | 1.0613 | No | No |
| I23 | 460 | Yes | ≥20 beds versus <5 beds | -7.1006 | 0.0301 | -7.2182 | 3.2645 | 0.0270 | -0.1176 | No | No |
| I23 | 460 | Yes | Evidence-based practice training: yes versus no | 3.9708 | 0.0243 | 4.0945 | 1.7906 | 0.0222 | 0.1236 | No | No |
| I23 | 460 | Yes | Microaspiration-prevention training: yes versus no | 3.9468 | 0.0224 | 4.3077 | 1.7565 | 0.0142 | 0.3609 | No | No |
| I24 | 461 | No | 10–14 beds versus <5 beds | -7.1610 | 0.0105 | -7.1735 | 2.8145 | 0.0108 | -0.0125 | No | No |
| I24 | 461 | No | 15–19 beds versus <5 beds | -8.3828 | 0.0171 | -9.3959 | 3.6027 | 0.0091 | -1.0131 | No | No |
| I24 | 461 | No | ≥20 beds versus <5 beds | -7.1006 | 0.0301 | -7.1151 | 3.2988 | 0.0310 | -0.0145 | No | No |
| I24 | 461 | No | Evidence-based practice training: yes versus no | 3.9708 | 0.0243 | 3.4219 | 1.7988 | 0.0571 | -0.5489 | No | Yes |
| I24 | 461 | No | Microaspiration-prevention training: yes versus no | 3.9468 | 0.0224 | 3.6109 | 1.7705 | 0.0414 | -0.3359 | No | No |
| I25 | 461 | Yes | 10–14 beds versus <5 beds | -7.1610 | 0.0105 | -7.1552 | 3.3483 | 0.0326 | 0.0058 | No | No |
| I25 | 461 | Yes | 15–19 beds versus <5 beds | -8.3828 | 0.0171 | -8.4104 | 4.0726 | 0.0389 | -0.0276 | No | No |
| I25 | 461 | Yes | ≥20 beds versus <5 beds | -7.1006 | 0.0301 | -6.7511 | 3.8294 | 0.0779 | 0.3494 | No | Yes |
| I25 | 461 | Yes | Evidence-based practice training: yes versus no | 3.9708 | 0.0243 | 3.8208 | 1.7765 | 0.0315 | -0.1500 | No | No |
| I25 | 461 | Yes | Microaspiration-prevention training: yes versus no | 3.9468 | 0.0224 | 4.1914 | 1.7469 | 0.0164 | 0.2446 | No | No |
| I26 | 461 | Yes | 10–14 beds versus <5 beds | -7.1610 | 0.0105 | -6.9390 | 3.3350 | 0.0375 | 0.2220 | No | No |
| I26 | 461 | Yes | 15–19 beds versus <5 beds | -8.3828 | 0.0171 | -7.9365 | 4.0444 | 0.0497 | 0.4463 | No | No |
| I26 | 461 | Yes | ≥20 beds versus <5 beds | -7.1006 | 0.0301 | -6.3849 | 4.2261 | 0.1308 | 0.7157 | No | Yes |
| I26 | 461 | Yes | Evidence-based practice training: yes versus no | 3.9708 | 0.0243 | 3.6092 | 1.7860 | 0.0433 | -0.3616 | No | No |
| I26 | 461 | Yes | Microaspiration-prevention training: yes versus no | 3.9468 | 0.0224 | 4.1469 | 1.7561 | 0.0182 | 0.2002 | No | No |
| I27 | 461 | Yes | 10–14 beds versus <5 beds | -7.1610 | 0.0105 | -7.1241 | 3.2620 | 0.0290 | 0.0369 | No | No |
| I27 | 461 | Yes | 15–19 beds versus <5 beds | -8.3828 | 0.0171 | -8.1609 | 3.9157 | 0.0371 | 0.2218 | No | No |
| I27 | 461 | Yes | ≥20 beds versus <5 beds | -7.1006 | 0.0301 | -7.0244 | 3.9188 | 0.0731 | 0.0762 | No | Yes |
| I27 | 461 | Yes | Evidence-based practice training: yes versus no | 3.9708 | 0.0243 | 3.5199 | 1.7741 | 0.0473 | -0.4509 | No | No |
| I27 | 461 | Yes | Microaspiration-prevention training: yes versus no | 3.9468 | 0.0224 | 3.7228 | 1.7459 | 0.0330 | -0.2240 | No | No |
| I28 | 461 | Yes | 10–14 beds versus <5 beds | -7.1610 | 0.0105 | -7.0220 | 3.2285 | 0.0296 | 0.1390 | No | No |
| I28 | 461 | Yes | 15–19 beds versus <5 beds | -8.3828 | 0.0171 | -8.2952 | 3.8801 | 0.0325 | 0.0876 | No | No |
| I28 | 461 | Yes | ≥20 beds versus <5 beds | -7.1006 | 0.0301 | -6.8349 | 3.8673 | 0.0772 | 0.2657 | No | Yes |
| I28 | 461 | Yes | Evidence-based practice training: yes versus no | 3.9708 | 0.0243 | 4.1469 | 1.8153 | 0.0223 | 0.1761 | No | No |
| I28 | 461 | Yes | Microaspiration-prevention training: yes versus no | 3.9468 | 0.0224 | 3.5261 | 1.7648 | 0.0457 | -0.4206 | No | No |
| I29 | 464 | Yes | 10–14 beds versus <5 beds | -7.1610 | 0.0105 | -7.8268 | 3.0392 | 0.0100 | -0.6658 | No | No |
| I29 | 464 | Yes | 15–19 beds versus <5 beds | -8.3828 | 0.0171 | -8.6506 | 3.6792 | 0.0187 | -0.2678 | No | No |
| I29 | 464 | Yes | ≥20 beds versus <5 beds | -7.1006 | 0.0301 | -5.6973 | 3.5836 | 0.1119 | 1.4033 | No | Yes |
| I29 | 464 | Yes | Evidence-based practice training: yes versus no | 3.9708 | 0.0243 | 3.4636 | 1.8003 | 0.0544 | -0.5072 | No | Yes |
| I29 | 464 | Yes | Microaspiration-prevention training: yes versus no | 3.9468 | 0.0224 | 3.9849 | 1.7593 | 0.0235 | 0.0381 | No | No |
| I30 | 464 | Yes | 10–14 beds versus <5 beds | -7.1610 | 0.0105 | -7.0809 | 3.2341 | 0.0286 | 0.0801 | No | No |
| I30 | 464 | Yes | 15–19 beds versus <5 beds | -8.3828 | 0.0171 | -8.1393 | 3.8991 | 0.0368 | 0.2435 | No | No |
| I30 | 464 | Yes | ≥20 beds versus <5 beds | -7.1006 | 0.0301 | -7.7553 | 3.9912 | 0.0520 | -0.6547 | No | Yes |
| I30 | 464 | Yes | Evidence-based practice training: yes versus no | 3.9708 | 0.0243 | 3.6991 | 1.7996 | 0.0398 | -0.2717 | No | No |
| I30 | 464 | Yes | Microaspiration-prevention training: yes versus no | 3.9468 | 0.0224 | 3.5081 | 1.7676 | 0.0472 | -0.4386 | No | No |
| I31 | 465 | Yes | 10–14 beds versus <5 beds | -7.1610 | 0.0105 | -7.7853 | 2.9923 | 0.0093 | -0.6243 | No | No |
| I31 | 465 | Yes | 15–19 beds versus <5 beds | -8.3828 | 0.0171 | -7.4641 | 3.6252 | 0.0395 | 0.9187 | No | No |
| I31 | 465 | Yes | ≥20 beds versus <5 beds | -7.1006 | 0.0301 | -9.0993 | 3.6519 | 0.0127 | -1.9987 | No | No |
| I31 | 465 | Yes | Evidence-based practice training: yes versus no | 3.9708 | 0.0243 | 3.8128 | 1.7818 | 0.0324 | -0.1581 | No | No |
| I31 | 465 | Yes | Microaspiration-prevention training: yes versus no | 3.9468 | 0.0224 | 3.9328 | 1.7545 | 0.0250 | -0.0140 | No | No |

**Note:** Each model was refitted after omission of one ICU. The analysis examined five core coefficients: the 10–14-bed, 15–19-bed, and ≥20-bed contrasts relative to <5 beds and the evidence-based practice training and microaspiration-prevention training coefficients. ΔB was calculated as the coefficient after cluster omission minus the corresponding full-sample coefficient. Direction change indicates a reversal in the sign of the coefficient, and significance change indicates a change in statistical significance at α=0.05. Models omitting I12 and I24 did not meet the convergence criterion. Estimates from models that did not meet the convergence criterion are presented for completeness but should not be interpreted as reliable inferential results. B, unstandardized coefficient; ICU, intensive care unit; LOCO, leave-one-cluster-out; SE, standard error.
