# Supplementary material for: Intensive Care Nurses' Self‐Reported Adherence to Evidence‐Informed Microaspiration‐Prevention Practices and Potential Implementation Determinants: A Multicenter Cross‐Sectional Study
Source: Nurs Open. 2026 Oct 21;13(10):e70914. doi: 10.1002/nop2.70914 (PMC13687589; doi:10.1002/nop2.70914)
Supplement: Supplementary file 2 [file NOP2-13-e70914-s002.docx]

**Supplementary File 1 - Source of Items** **in the Adherence to Microaspiration Prevention**

| **Domain** | **Item** | **References** |
| --- | --- | --- |
| Basic Nursing | 1.Elevate the head of bed to 30°-45° for intubated patients to reduce the risk of microaspiration.^a^ | (Alanazi & Alonazi, 2024; Alhamad & Elsayed, 2024; Barnawi et al., 2025; Chen et al., 2023; Deng et al., 2024; Deng et al., 2023; Kang & Zhou, 2024; Pozuelo-Carrascosa et al., 2022; Xiao et al., 2023; Zhang, 2021) |
|  | 2.Reposition the patient every 1-2 hours.^c^ | (Alanazi & Alonazi, 2024; Alhamad & Elsayed, 2024; Deng et al., 2023; Qin et al., 2026; Zhang, 2021) |
|  | 3.Perform oral care for the patient at least twice daily.ᵃ˒ᵈ | (Alanazi & Alonazi, 2024; Alhamad & Elsayed, 2024; Deng et al., 2024; Deng et al., 2023; Ehrenzeller & Klompas, 2023; Klompas et al., 2022; Xiao et al., 2023; Zhang, 2021) |
|  | 4.Use a combined oral care approach involving tooth brushing, rinsing, and suctioning.ᵃ˒ᶜ | (Deng et al., 2024; Deng et al., 2023; Xiao et al., 2023; Zhang, 2021; Zhao et al., 2020) |
| Cuff Management | 5. Maintain the cuff pressure between 25-30 cmH₂O.ᵃ | (Alanazi & Alonazi, 2024; Chen et al., 2023; Deng et al., 2024; Deng et al., 2023; Kang & Zhou, 2024; Lizy et al., 2014; Maertens et al., 2022; Xiao et al., 2023; Zhang, 2021) |
|  | 6. Select high-volume, low-pressure or tapered cuff endotracheal tubes.ᵈ | (Chen et al., 2023; D'haese et al., 2013; Deng et al., 2024; Deng et al., 2023; Kang & Zhou, 2024; Xiao et al., 2023; Zhang, 2021) |
|  | 7.Clear the secretion above the cuff every 2 hours.ᵇ˒ᵈ | (Alhamad & Elsayed, 2024; Chen et al., 2023; Deng et al., 2024; Deng et al., 2023; Hu & Tursuniyazi, 2026; Xiao et al., 2023; Zhang, 2021) |
|  | 8. Monitor and record the cuff pressure every 4-6 hours.ᵈ | (Chen et al., 2023; Deng et al., 2024; Deng et al., 2023; Kang & Zhou, 2024; Lizy et al., 2014; Maertens et al., 2022; Xiao et al., 2023; Zhang, 2021) |
|  | 9. Use a manometer to monitor the cuff pressure instead of the tactile method.ᵃ | (Ban et al., 2023; Deng et al., 2024; Deng et al., 2023; Fernandez et al., 1990; Xiao et al., 2023; Zhang, 2021) |
|  | 10. Verify the cuff pressure is appropriate before performing oral care.ᶜ | (Beccaria et al., 2017; Deng et al., 2023; Goodfellow et al., 2024; Haghighi et al., 2017; Xiao et al., 2023; Zhang, 2021) |
| Subglottic Suctioning | 11. Recommend that the physician use an endotracheal tube with subglottic drainage capability, if possible.ᵃ˒ᵇ | (Alanazi & Alonazi, 2024; Alhamad & Elsayed, 2024; Chen et al., 2023; Deng et al., 2023; Hu & Tursuniyazi, 2026; Klompas et al., 2022; Xiao et al., 2023) |
|  | 12.Perform regular subglottic suctioning to clear the patient's secretions.ᵃ˒ᵇ | (Alhamad & Elsayed, 2024; Barnawi et al., 2025; Chen et al., 2023; Deng et al., 2024; Deng et al., 2023; Hu & Tursuniyazi, 2026; Xiao et al., 2023; Zhang, 2021) |
|  | 13. Implement subglottic secretion drainage to reduce the risk of microaspiration.ᵃ˒ᵇ | (Alanazi & Alonazi, 2024; Alhamad & Elsayed, 2024; Chen et al., 2023; Deng et al., 2024; Deng et al., 2023; Hu & Tursuniyazi, 2026; Xiao et al., 2023; Zhang, 2021) |
| Enteral Nutrition | 14. Assess the patient's gastric residual volume and adjust the enteral feeding rate accordingly.ᵇ˒ᵈ | (Barnawi et al., 2025; Feng et al., 2023; McClave et al., 2016; Miao et al., 2023; Singer et al., 2019; Xiao et al., 2023; Zhang, 2021; Zou et al., 2024) |
|  | 15. Assess and adjust the method, rate, or position of enteral nutrition as needed.ᵃ˒ᵇ | (McClave et al., 2016; Miao et al., 2023; Singer et al., 2019; Xiao et al., 2023; Zhang, 2021; Zou et al., 2024) |
|  | 16. Adjust the patient's nutritional volume and rate to minimize gastric distension.ᵇ˒ᶜ | (McClave et al., 2016; Singer et al., 2019; Xiao et al., 2023; Zhang, 2021) |
|  | 17. Assess the cuff pressure before nasogastric feeding, followed by suctioning of secretions and gastric contents.ᶜ˒ᵈ | (Deng et al., 2024; Deng et al., 2023; Seliman et al., 2011; Xiao et al., 2023; Zhang, 2021; Zou et al., 2024) |
|  | 18.Administer feeding using either intermittent bolus or continuous infusion methods.ᵈ | (Heffernan et al., 2022; Singer et al., 2019; Xiao et al., 2023; Zhang, 2021) |
| Risk Management | 19.Have the ability to identify patients at high risk of microaspiration.ᶜ | (Deng et al., 2024; Deng et al., 2023; Kang & Zhou, 2024; Klompas et al., 2022; Xiao et al., 2023; Zhang, 2021) |
|  | 20.Perform continuous risk assessment for microaspiration in high-risk patients during nursing care.ᶜ | (Deng et al., 2024; Deng et al., 2023; Kang & Zhou, 2024; Klompas et al., 2022; McClave et al., 2016; Xiao et al., 2023; Zhang, 2021; Zou et al., 2024) |
|  | 21. Collaborate with a multidisciplinary team to manage microaspiration collectively.ᶜ | (Alanazi & Alonazi, 2024; Klompas et al., 2022; Zhang, 2021) |
|  | 22. Have the ability to use or assist in using bedside gastric ultrasound to assess the patient's gastric residual volume（GRV）, if necessary.ᵇ˒ᵈ | (Brotfain et al., 2022; Pan et al., 2024; Xiao et al., 2023) |

**Note:** Superscripts indicate the context of the supporting evidence rather than a formal grading of evidence quality. ᵃGuideline-supported practice, although the certainty or specificity of the supporting evidence may vary. ᵇCondition-specific practice applicable to selected patients or when clinically indicated. ᶜSupported mainly by multicomponent bundles, clinical protocols, expert guidance, or indirect evidence; its independent effect has not been established. ᵈMixed or limited evidence regarding the specific procedure, frequency, or effectiveness as a stand-alone intervention. The cited references indicate the sources and clinical rationale used to develop the questionnaire items and should not be interpreted as demonstrating that every individual practice independently reduces microaspiration or ventilator-associated pneumonia. Citations to Zou et al. (2024) and Kang and Zhou (2024) indicate their use as literature sources supporting the clinical rationale for relevant items and do not indicate any sharing of questionnaire responses, participants, or datasets.
