# Supplementary material for: Intensive Care Nurses' Self‐Reported Adherence to Evidence‐Informed Microaspiration‐Prevention Practices and Potential Implementation Determinants: A Multicenter Cross‐Sectional Study
Source: Nurs Open. 2026 Oct 21;13(10):e70914. doi: 10.1002/nop2.70914 (PMC13687589; doi:10.1002/nop2.70914)
Supplement: Supplementary file 3 [file NOP2-13-e70914-s003.docx]

**Supplementary File 2 - Adherence to the Microaspiration Prevention (N=476)**

| **Domain** | **Item** | Mean (SD) | Never  n (%) | Rarely  n (%) | Sometimes  n (%) | Often  n (%) | Always  n (%) |
| --- | --- | --- | --- | --- | --- | --- | --- |
| Basic Nursing | 1.Elevate the head of bed to 30°-45° for intubated patients to reduce the risk of microaspiration. | 3.68±1.35 | 50(10.5） | 56(11.76) | 65(13.66) | 131(27.52) | 174(36.55) |
|  | 2.Reposition the patient every 1-2 hours. | 3.67±1.31 | 47(9.87） | 51(10.71) | 79(16.6) | 135(28.36) | 164(34.45) |
|  | 3.Perform oral care for the patient at least twice daily. | 3.70±1.37 | 55(11.55） | 48(10.08) | 63(13.24) | 129(27.1) | 181(38.03) |
|  | 4.Use a combined oral care approach involving tooth brushing, rinsing, and suctioning. | 3.68±1.34 | 48(10.08) | 59(12.39) | 66(13.87) | 128(26.89) | 175(36.76) |
| Cuff Management | 5. Maintain the cuff pressure between 25-30 cmH₂O. | 3.50±1.37 | 64(13.45) | 51(10.71) | 87(18.28) | 132(27.73) | 142(29.83) |
|  | 6. Select high-volume, low-pressure or tapered cuff endotracheal tubes. | 3.51±1.34 | 56(11.76） | 65(13.66) | 69(14.5) | 150(31.51) | 136(28.57) |
|  | 7.Clear the secretion above the cuff every 2 hours. | 3.46±1.40 | 68(14.29） | 63(13.24) | 62(13.03) | 147(30.88) | 136(28.57) |
|  | 8. Monitor and record the cuff pressure every 4-6 hours. | 3.45±1.40 | 72(15.13） | 57(11.97) | 68(14.29) | 143(30.04) | 136(28.57) |
|  | 9. Use a manometer to monitor the cuff pressure instead of the tactile method. | 3.52±1.36 | 56(11.76） | 68(14.29) | 66(13.87) | 145(30.46) | 141(29.62) |
|  | 10. Verify the cuff pressure is appropriate before performing oral care. | 3.54±1.41 | 62(13.03） | 66(13.87) | 62(13.03) | 126(26.47) | 160(33.61) |
| Subglottic Suctioning | 11. Recommend that the physician use an endotracheal tube with subglottic drainage capability, if possible. | 3.46±1.38 | 60(12.61） | 73(15.34) | 68(14.29) | 138(28.99) | 137(28.78) |
|  | 12.Perform regular subglottic suctioning to clear the patient's secretions. | 3.38±1.39 | 71(14.92） | 65(13.66) | 75(15.76) | 142(29.83) | 123(25.84) |
|  | 13. Implement subglottic secretion drainage to reduce the risk of microaspiration. | 3.47±1.44 | 73(15.34） | 62(13.03) | 62(13.03) | 127(26.68) | 152(31.93) |
| Enteral Nutrition | 14. Assess the patient's gastric residual volume and adjust the enteral feeding rate accordingly. | 3.58±1.38 | 59(12.39） | 58(12.18) | 69(14.5) | 128(26.89) | 162(34.03) |
|  | 15. Assess and adjust the method, rate, or position of enteral nutrition as needed. | 3.53±1.30 | 48(10.08) | 67(14.08) | 74(15.55) | 157(32.98) | 130(27.31) |
|  | 16. Adjust the patient's nutritional volume and rate to minimize gastric distension. | 3.44±1.34 | 65(13.66) | 61(12.82) | 65(13.66) | 171(35.92) | 114(23.95) |
|  | 17. Assess the cuff pressure before nasogastric feeding, followed by suctioning of secretions and gastric contents. | 3.56±1.40 | 66(13.87) | 55(11.55) | 56(11.76) | 143(30.04) | 156(32.77) |
|  | 18.Administer feeding using either intermittent bolus or continuous infusion methods. | 3.59±1.33 | 56(11.76) | 47(9.87) | 80(16.81) | 148(31.09) | 145(30.46) |
| Risk Management | 19.Have the ability to identify patients at high risk of microaspiration. | 3.37±1.46 | 82(17.23) | 64(13.45) | 61(12.82) | 132(27.73) | 137(28.78) |
|  | 20.Perform continuous risk assessment for microaspiration in high-risk patients during nursing care. | 3.33±1.46 | 88(18.49) | 59(12.39) | 69(14.5) | 130(27.31) | 130(27.31) |
|  | 21. Collaborate with a multidisciplinary team to manage microaspiration collectively. | 3.41±1.41 | 70(14.71) | 71(14.92) | 65(13.66) | 134(28.15) | 136(28.57) |
|  | 22. Have the ability to use or assist in using bedside gastric ultrasound to assess the patient's gastric residual volume（GRV）, if necessary. | 3.35±1.45 | 78(16.39) | 74(15.55) | 62(13.03) | 128(26.89) | 134(28.15) |
